# Discovery of a silicate rock-boring organism and macrobioerosion in fresh water

**DOI:** 10.1038/s41467-018-05133-4

**Published:** 2018-07-23

**Authors:** Ivan N. Bolotov, Olga V. Aksenova, Torkild Bakken, Christopher J. Glasby, Mikhail Yu. Gofarov, Alexander V. Kondakov, Ekaterina S. Konopleva, Manuel Lopes-Lima, Artyom A. Lyubas, Yu Wang, Andrey Yu. Bychkov, Agniya M. Sokolova, Kitti Tanmuangpak, Sakboworn Tumpeesuwan, Ilya V. Vikhrev, J. Bruce H. Shyu, Than Win, Oleg S. Pokrovsky

**Affiliations:** 1 Northern Arctic Federal University, Arkhangelsk, Russia 163002; 2 Federal Center for Integrated Arctic Research, Russian Academy of Sciences, Arkhangelsk, Russia 163000; 3 Norwegian University of Science and Technology, NTNU University Museum, 7491 Trondheim, Norway; 4 Museum and Art Gallery of the Northern Territory, Darwin, NT 0820 Australia; 5 CIIMAR—Interdisciplinary Centre of Marine and Environmental Research, University of Porto, 4450-208 Matosinhos, Portugal; 6 CIBIO/InBIO—Research Center in Biodiversity and Genetic Resources, University of Porto, 4485-661 Vairão, Portugal; 7 Earth Observatory of Singapore, Nanyang Technological University, Singapore, 639798 Singapore; 8 Department of Geosciences, National Taiwan University, Taipei, 10617 Taiwan; 9 Faculty of Geology, Lomonosov Moscow State University, Moscow, Russia 119991; 10 V. I. Vernadsky Institute of Geochemistry and Analytical Chemistry, Russian Academy of Sciences, Moscow, Russia 119991; 11 A. N. Severtsov Institute of Ecology and Evolution, Russian Academy of Sciences, Moscow, Russia 119071; 12 N. K. Koltzov Institute of Developmental Biology, Russian Academy of Sciences, Moscow, Russia 119991; 13 Department of Science, Faculty of Science and Technology, Loei Rajabhat University, Loei, 42000 Thailand; 14 Department of Biology, Faculty of Science, Mahasarakham University, Maha Sarakham, 44150 Thailand; 15 Department of Zoology, Hpa-An University, Hpa-An, Kayin State 13017 Myanmar; 16 Geosciences and Environment Toulouse, UMR 5563 CNRS, 31400 Toulouse, France; 17 BIO-GEO-CLIM Laboratory, Tomsk State University, Tomsk, Russia 634050

## Abstract

Macrobioerosion is a common process in marine ecosystems. Many types of rock-boring organisms break down hard substrates, particularly carbonate rocks and calcareous structures such as dead corals and shells. In paleontology, the presence of rocks with boreholes and fossil macroboring assemblage members is one of the primary diagnostic features of shallow marine paleo-environments. Here we describe a silicate rock-boring organism and an associated community in submerged siltstone rock outcrops in Kaladan River, Myanmar. The rock-boring mussel *Lignopholas fluminalis* is a close relative of the marine piddocks, and its borings belong to the ichnospecies *Gastrochaenolites anauchen*. The neotectonic uplift of the area leading to gradual decrease of the sea level with subsequent shift from estuarine to freshwater environment was the most likely driver for the origin of this community. Our findings highlight that rocks with macroborings are not an exclusive indicator of marine paleo-ecosystems, but may also reflect freshwater habitats.

## Abstract (web summary)

Macrobioerosion, the boring of rock and other hard substrates by living organisms, is used as a marker of marine paleo-environments. Here, Bolotov et al. describe a rock-boring mussel and its associated community from freshwater in Myanmar, demonstrating that macrobioerosion is a wider phenomenon.

## Introduction

Bioerosion is a process by which a living organism incises or bores different hard substrates (e.g. rocks, shells, corals, and bones) by mechanical disruption and/or chemical decomposition^1^. The eroding activities of living organisms are important factors in marine sedimentation and benthic ecology^2^. Bioerosion increases species diversity of marine hard substrate communities by increasing habitat complexity or as a result of the increase in accessible surface area for colonization^3^. Additionally, this process influences the evolution of coastal profiles over long timescales^4,5^. With respect to their environmental impact, erosional organisms represent an important group of ecosystem engineers^6,7^.

The investigation of bioerosion patterns supplies significant background information to other research areas (e.g., zoology, paleoecology, biogeochemistry, sedimentology, and biostratigraphy)^1,8^. Macrobioerosion trace fossils are important indicators of intertidal and shallow subtidal marine paleo-environments^9–11^, e.g., boring bivalves may be used as biological sea level indicators marking ancient shorelines^2,11,12^. Boring organisms have a great economic impact because they damage ships, fishing equipment, archeological heritage, various infrastructures in marine environments, and the shells of aquaculture molluscs^12–14^.

Rock borers occur in a broad range of taxonomic groups, including bivalves, gastropods, polychaetes and sipunculans (Annelida), sea urchins, sponges, and others^8–10,15^. The piddocks, or rock-boring mussels (Mollusca: Bivalvia: Pholadidae), are predominantly marine animals specialized for boring into a plethora of available substrates such as soft silicate and carbonate rocks, clay, corals, wood, and peat^3,14,16^. The genera *Lignopholas* Turner, 1955 and *Martesia* Sowerby 1824 contain several estuarine and marine wood-boring bivalve species, including a single species that can bore into living mangrove trees^17^. There is a unique record of clavate borings and fossil specimens of *Martesia* sp. from Cretaceous Burmese amber^11^. However, *Lignopholas fluminalis* was also collected from soft argillite rocks and brickworks in brackish sections of rivers in India and Myanmar^17–19^. The piddocks have developed a variety of adaptations to accommodate their rock-boring behavior and make their borings mechanically, by scraping at the substratum^20^.

In the marine environment, almost all macroborings are recorded in calcareous substrates, e.g., corals, shells, and carbonate rocks^10,21^. There are far fewer examples of fossil and recent marine borings found in non-calcareous rocks and minerals such as siltstones, quartzites, basalts, andesites, dolerites, gneisses, and others^17,21,22^. From an ichnological point of view, clavate macroborings in hardgrounds and in fossil wood belong to two ichnogenera, i.e., *Gastrochaenolites* Leymerie 1842 and *Teredolites* Leymerie 1842 for borings in lithic and lignic substrates, respectively^23^. The *Gastrochaenolites* borings are known from the Early Ordovician to Recent and they are mainly produced by marine endolithic bivalves^8,9^. However, several recent gastropods and sipunculan worms may also produce *Gastrochaenolites*^9^.

In general, macrobioerosion was considered an exclusively marine and, to a lesser degree, an estuarine process^8,10^. However, we discovered the macrobioerosion of silicate siltstone rocks caused by a rock-boring mussel species in a freshwater section of the Kaladan River, Myanmar, which greatly expands our knowledge of bioerosion. Our phylogenetic and biogeographic modeling shows that at least three members of this rock-boring assemblage are relict marine-derived lineages, which emphasizes a broad-scale expansion of saltwater taxa into freshwater environment in Southeast Asia.

## Results

### Discovery of freshwater macrobioerosion

**Fig. 1:**
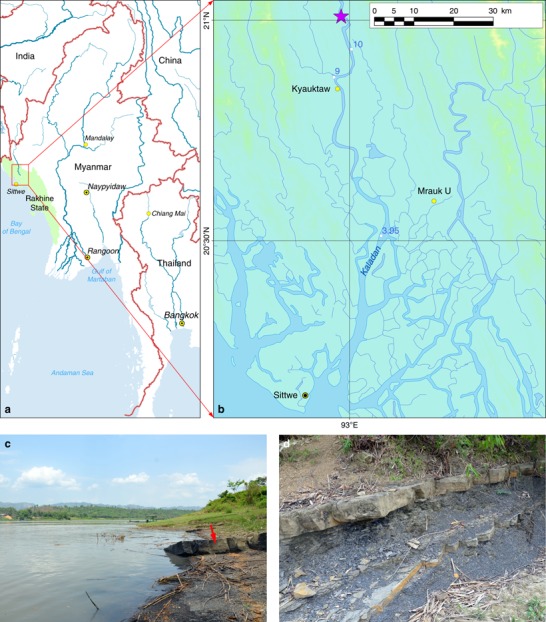
Freshwater bioerosion site, Kaladan River, Myanmar. **a** Map of Myanmar with location of the downstream area of Kaladan River (red frame). **b** Map of the study site. The violet asterisk indicates the freshwater rock-borer’s site, and white circles indicate the levels of river water above sea level. The yellow circles indicate main towns. The maps were created using ESRI ArcGIS 10 software (www.esri.com/arcgis); the topographic base of the maps was created with Natural Earth Free Vector and Raster Map Data (www.naturalearthdata.com), Vector Map (VMap) Level 0 (http://earth-info.nga.mil/publications/vmap0.html), and ASTER GDEM (https://lpdaac.usgs.gov/node/1079) (Maps: Mikhail Yu. Gofarov). **c** River site with freshwater rock-borer’s ecosystem. The red arrow indicates siltstone rocks, a substrate of rock-boring bivalves. **d** Fragment of the black siltstone outcrop, the submerged part of which forms the basis of freshwater rock-borer ecosystem. (Photos: Olga V. Aksenova)

The site is located in the middle reaches of Kaladan River: 21.0094° N, 92.9813° E, altitude of 11 m a.s.l., Rakhine State, western Myanmar (Fig. 1a, b). The rock-boring assemblage is associated with submerged black siltstone rocks located in the lower part and bottom of the river valley, with an above-water outcrop near the bioerosion site (Fig. 1b–d). The submerged rocks are located within a riverine section of ~500 m in length and of ~300 m in width, with depth values of 1–2 m and more.

### Freshwater environment

**Fig. 2:**
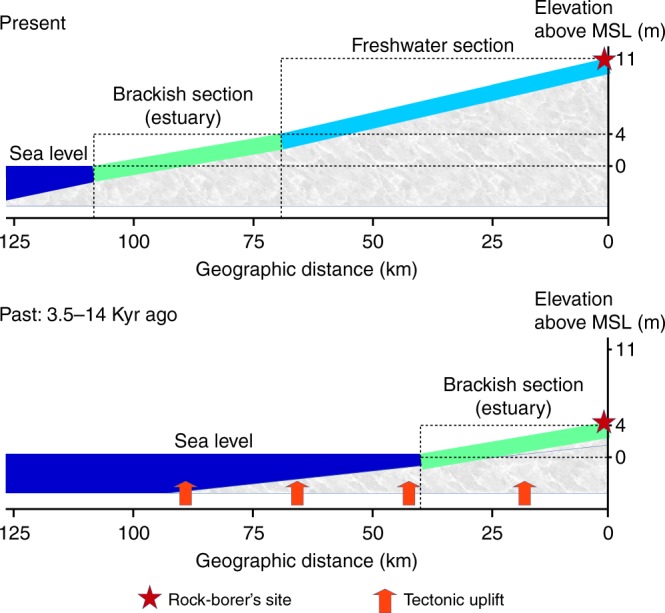
Model of neotectonic evolution of the lower course of the Kaladan River. The long-term uplift rate *R* of 0.5–2 mm/year was applied (see Methods section). The model assumes a gradual shift from estuarine to freshwater environment at the rock-borer’s site due to the tectonic uplift with subsequent adaptation of the population of the brackish rock-boring species *Lignopholas fluminalis* to freshwater habitat. (Model: Ivan N. Bolotov, Mikhail Yu. Gofarov, Yu Wang, & J. Bruce H. Shyu)

The model of tidal influence reveals that the distance between the rock-borer’s site and the upper level of the Kaladan estuary is 71 km (Fig. 2). Dating of the shift from estuarine to freshwater environment at the site suggests that it was approximately 3.5–14 Kyr ago, i.e., not earlier than the Late Pleistocene (Fig. 2). The analysis of a water sample confirms that it is a freshwater section of the river, with a chloride ion concentration of 2.7 mg/L, sodium ion concentration of 11.6 mg/L, and a total salinity of 0.16‰ (Supplementary Table 1).

### Rock substrate

According to our grain-size analyses (Supplementary Fig. 1), the substrate belongs to siltstone (aleurolite) rocks due to a primary grain size of 2–62 µm. The backscattered electron (BSE) images and X-ray diffraction (XRD) analyses indicate that the rock consists of quartz, feldspar, clay minerals, chlorite, and mica grains (Supplementary Table 2 and Supplementary Fig. 1). The X-ray fluorescence (XRF) analysis reveals the dominance of silicon dioxide (56.9 ± 0.5%) and aluminum oxide (17.0 ± 0.4%) (Supplementary Table 3). A mean microindentation hardness (Vickers test) value of the substrate rock is 0.62 GPa with a range of 0.50–0.72 GPa (mean HV value = 61.9 kgf × mm^−2^, range = 52.1–73.5 kgf × mm^−2^).

### Ichnotaxonomy

**Fig. 3:**
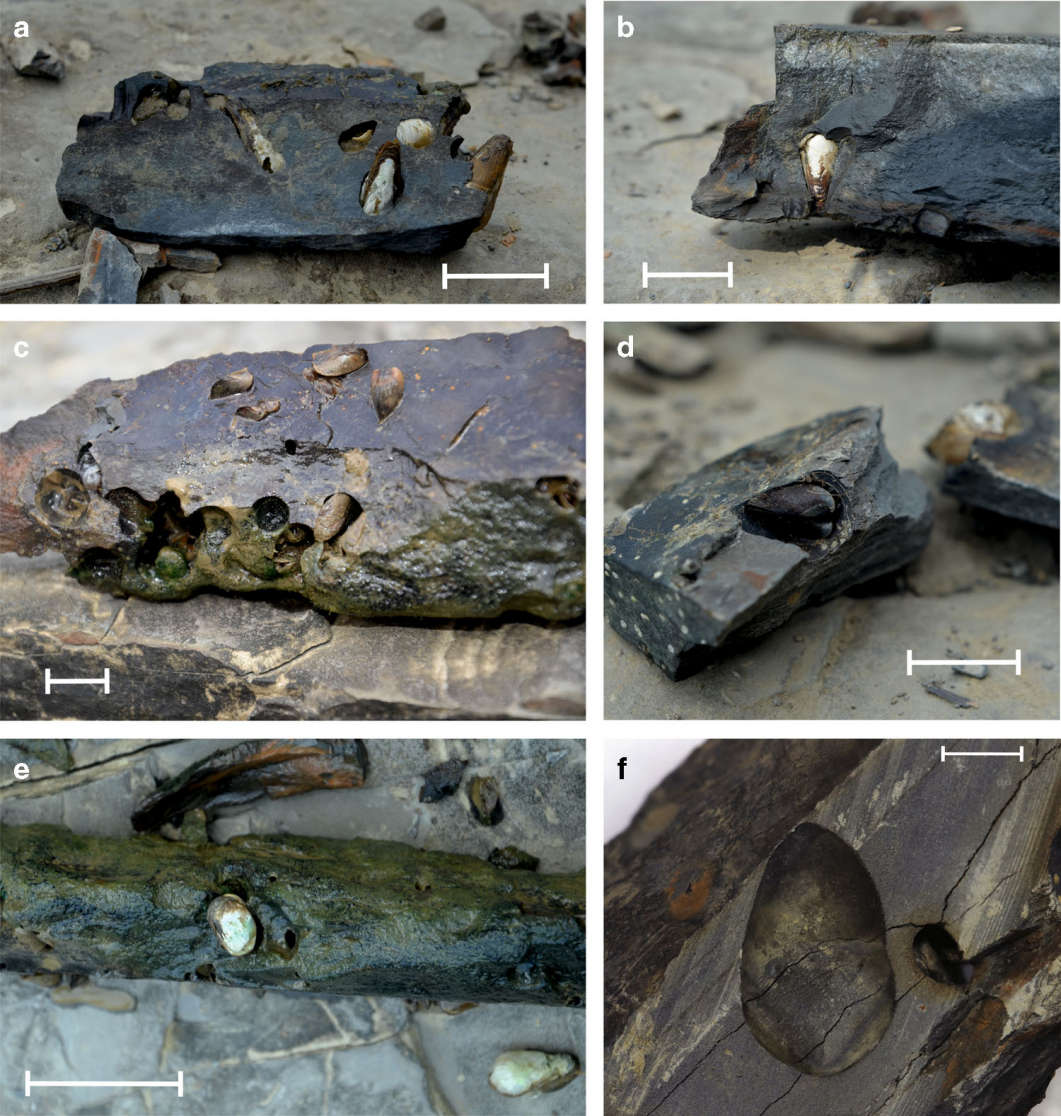
Blocks of siltstone rocks with borings and living macroinvertebrates from the freshwater bioerosion site at the Kaladan River, Myanmar. **a**, **b** Rock-boring mussels, *Lignopholas fluminalis*, in their clavate borings (ichnospecies: *Gastrochaenolites anauchen*). **c** Arc clams, *Scaphula deltae*, and empty borings filled with clay, a habitat of polychaetes *Neanthes meggitti* and *Namalycastis indica* (Nereididae). **d** Arc clam, *Scaphula deltae*, in an empty boring. **e** Jackknife clam, *Novaculina gangetica*, and sponge, *Corvospongilla ultima*. Scale bars = 10 mm (Photos: Olga V. Aksenova). **f** Longitudinal cross-section of the boring, *Gastrochaenolites anauchen*. Scale bar = 5 mm. (Photo: Ilya V. Vikhrev)

The macroborings from the Kaladan River are clavate, circular in cross-section throughout the length, expanded gradually below the aperture, with a greatest diameter about three-fourths of the depth; bases are rounded; sides are smooth, without clear circular or spiral bioglyph; a distinguishable neck is lacking (Fig. 3f). With respect to the lithic substrate and morphological features^24^, these borings correspond well to the ichnospecies *Gastrochaenolites anauchen* Wilson & Palmer, 1998.

### Rock-boring species

**Fig. 4:**
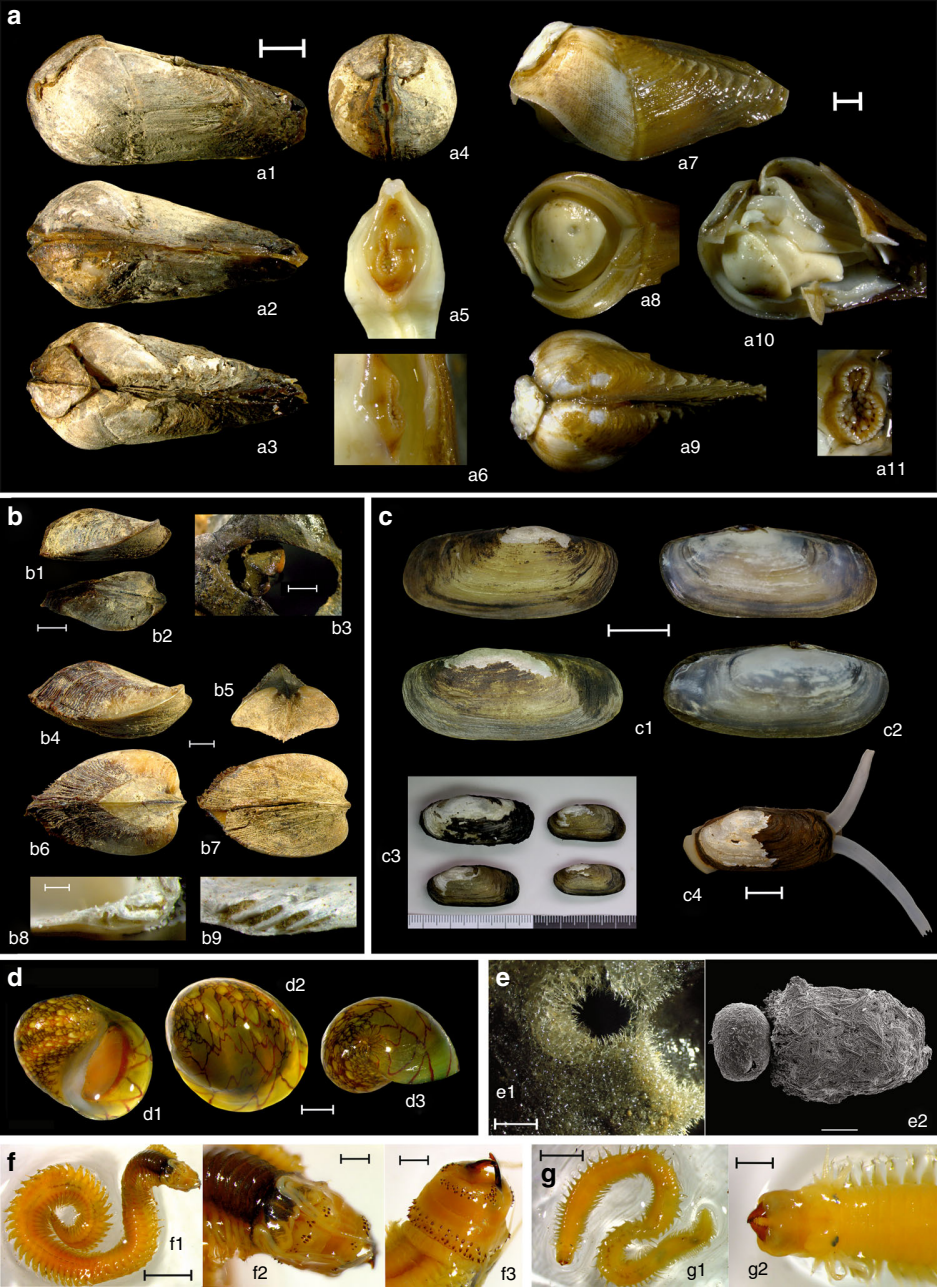
Rock-boring and nestling macroinvertebrates associated with freshwater bioerosion site at the Kaladan River, western Myanmar. **a** *Lignopholas fluminalis*, a rock-boring species: (a1) Lateral view of an adult specimen with fully developed callum, (a2) Ventral view of apposed valves, (a3) Dorsal view of apposed valves, (a4) Anterior view, (a5) Frontal view of siphons, (a6) Lateral view of siphons, (a7) Lateral view of a young specimen, (a8) Ventral view of the anterior end, (a9) Dorsal view, (a10) Internal morphology of soft body, (a11) Frontal view of siphons (scale bars = 2 mm). **b** *Scaphula deltae*, an ark clam species: (b1) Lateral and (b2) dorsal view of a young specimen, (b3) Live clam attached by byssus in borehole, (b4) Lateral and (b5) frontal view of an adult specimen, (b6) Dorsal and (b7) ventral view of an adult specimen, (b8) Cardinal teeth of left valve, (b9) Cardinal teeth of right valve (scale bars = 2 mm). **c** *Novaculina gangetica*, a jackknife clam species: (c1) Lateral view of right and left shell valves (outside), (c2) Lateral view of right and left shell valves (inside), (c3) Shell variability, (c4) Live specimen with protruding foot and siphons (scale bars = 10 mm). **d** *Clithon* cf. *reticularis*, a gastropod species: (d1) Apertural view, (d2) Dorsal view, (d3) Apical view (scale bars = 2 mm). (**e**) *Corvospongilla ultima*, a sponge species: (e1) General view of a sponge body fragment (scale bar = 2 mm), (e2) Three adherent gemmules; gemmular cage covering the left gemmule is removed (scale bar = 500 µm). **f** *Neanthes meggitti*, a polychaete species: (f1) Dorsal view of complete specimen (scale bar = 2 mm), (f2) Dorsal view of the anterior end (scale bar = 0.5 mm), (f3) Ventral view of the anterior end (scale bar = 0.5 mm). **g** *Namalycastis indica*, a polychaete species: (g1) Dorsal view of animal (scale bar = 2 mm), (g2) Dorsal view of the anterior end (scale bar = 0.5 mm). (Photos: Olga V. Aksenova [**a**, **b**, **d**, e1, **f**, and **g**], Ekaterina S. Konopleva and Ilya V. Vikhrev [**c**], and Agniya M. Sokolova [e2])

**Fig. 5:**
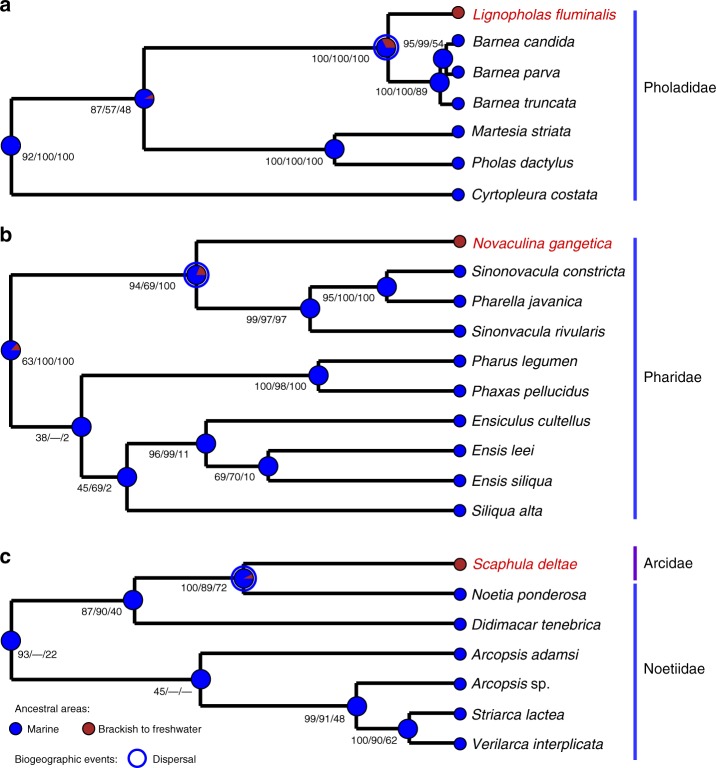
Ultrametric Bayesian (BEAST 2) phylogenies showing the primary marine origin of rock-boring and nestling bivalve species from the bioerosion site in the Kaladan River, including **a** *Lignopholas fluminalis*, Pholadidae (*18S rRNA* + *28S rRNA*, 3033 bp), **b** *Novaculina gangetica*, Pharidae (*COI* + *16S rRNA* + *28S rRNA*, 2602 bp), and **b** *Scaphula deltae*, Arcidae (*28S rRNA* + *18S rRNA*, 2450 bp, full tree includes 44 taxa but the other clade of Arcidae is not shown here: see Supplementary Fig. 2). Black numbers near nodes are BPP values inferred from BEAST/BPP values inferred from MrBayes/BS values inferred from RAxML (“—” indicates a topological difference). Pie chaps on the nodes indicate the probabilities of certain ancestral areas with respect to combined results under three different models (S-DIVA, DEC, and S-DEC) inferred from RASP v. 3.2

Freshwater rock borers heavily invaded the submerged section of siltstone rocks that have numerous piddock-like boreholes (Fig. 3a–f). Empty borings are sometimes filled with clay (Fig. 3c). This boring species was determined as *Lignopholas fluminalis* on the basis of morphological characters (Table 1, Fig. 4a and Supplementary Note 1). The *COI* gene sequences indicates that it is related to the marine piddock species *Barnea davidi* but with low similarity (81.4%), while the nuclear *18S rRNA* gene sequences show a very close relationship with several other marine *Barnea* spp. (similarity of 98–99%) (Supplementary Table 4). The Bayesian and maximum likelihood phylogenies reveal that this species is a sister lineage of *Barnea* spp. The ancestral area reconstruction models suggest that the most recent common ancestor (MRCA) of the *Lignopholas* + *Barnea* clade was a marine mussel (Fig. 5a and Table 2). The boring mechanism of *L. fluminalis* is not known, but their boreholes should be made mechanically as do its marine relatives^20^.

**Table 1:** Taxonomy, ecology, and reference sequences of freshwater rock-borer community’s members from Kaladan River, western Myanmar

Phylum: Class: Family	Species	Role in the community, preferred environment	Origin of the family	Acc. numbers of reference sequences			
				*COI*	*16S rRNA*	*28S rRNA*	*18S rRNA*
Mollusca: Bivalvia: Pholadidae	*Lignopholas fluminalis* (Blanford, 1867)	Rock-boring species, brackish	Marine^14^	MF958974	n/a	n/a	n/a
				MF958975	n/a	n/a	MF959022
				MF958976	n/a	n/a	MF959023
				MF958977	n/a	n/a	MF959024
				MF958978	n/a	n/a	MF959025
				MF958979	n/a	n/a	MF959026
Mollusca: Bivalvia: Arcidae	*Scaphula deltae* (Blanford, 1867)	Nestling species, from brackish to freshwater	Marine^73^	MF958980	n/a	n/a	n/a
				MF958981	n/a	MF959007	n/a
				MF958982	n/a	n/a	n/a
				MF958983	n/a	MF959008	n/a
				MF958984	n/a	MF959009	n/a
				MF958985	n/a	MF959010	n/a
Mollusca: Bivalvia: Pharidae	*Novaculina gangetica* (Benson, 1830)	Nestling species, from brackish to freshwater	Marine^74^	MF958986 *	MF958997 *	MF959011 *	n/a
				MF958987 *	MF958998 *	MF959012 *	n/a
				MF958988 *	MF958999 *	MF959013 *	n/a
				MF958989	MF959000	MF959000x	n/a
				MF958990	MF959001	MF959015	n/a
				MF958991	MF959002	MF959016	n/a
Mollusca: Gastropoda: Neritidae	*Clithon* cf. *reticularis* (Sowerby, 1838)	Nestling species, probably brackish	Marine^75^	MF958992	MF959003	MF959017	n/a
				MF958993	MF959004	MF959018	n/a
Annelida: Phyllodocida: Nereididae	*Neanthes meggitti* (Monro, 1931)	Nestling species, brackish	Marine^76^	MF958994	MF959006	MF959020	n/a
Annelida: Phyllodocida: Nereididae	*Namalycastis indica* (Southern, 1921)	Nestling species, freshwater	Marine^76^	MF958995	MF959005	MF959019	n/a
				MG759522	MG759523	MG759524	n/a
Porifera: Demospongiae: Spongillidae	*Corvospongilla ultima* (Annandale, 1910)	Nestling species, freshwater	Freshwater^77^	MF958996	n/a	MF959021	n/a

n/a—not available

^*^Specimens that were collected outside the rock-borer site, from another river (Myanmar: Lemro River, 20.6150° N, 93.2481° E)

**Table 2:** Evolutionary origin of bivalve lineages from the freshwater rock-borer community in western Myanmar on the basis of ancestral area reconstruction analyses

MRCA	The most likely ancestral area	Biogeographic event (origin of estuarine/freshwater lineages)	Probability of the most likely ancestral area			
			S-DIVA	DEC	S-DEC	Combined results
*Lignopholas* + *Barnea*	Marine	Dispersal	1.00	0.52	0.52	0.68
*Novaculina* + (*Pharella* + *Sinonovacula*)	Marine	Dispersal	1.00	0.64	0.79	0.81
*Scaphula* + *Noetia*	Marine	Dispersal	1.00	0.87	0.90	0.92

### Associated nestling species

We recorded six species of nestling macroinvertebrates that appear to be associated with this rock-borer’s ecosystem (Table 1, Supplementary Table 4, and Supplementary Note 1). An integrative study of the species using morphological and molecular approaches indicates that they include two clams, *Scaphula deltae* (Arcidae) and *Novaculina gangetica* (Pharidae), a gastropod, *Clithon* cf. *reticularis* (Neritidae), two polychaetes, *Namalycastis indica* and *Neanthes meggitti* (Nereididae), and a sponge, *Corvospongilla ultima* (Spongillidae) (Fig. 4b–g). The results of phylogenetic and biogeographic modeling reveal that both clam taxa, *S. deltae* and *N. gangetica*, most likely represent the relict marine-derived freshwater lineages (Fig. 5b, c and Table 2). *N. gangetica* is a close relative of the *Sinonovacula* + *Pharella* clade (Fig. 5b and Supplementary Table 4). *S. deltae* clusters together with several members of the Noetiidae but not with the Arcidae (Fig. 5c, Supplementary Fig. 2, and Supplementary Table 4).

## Discussion

Here we present the discovery of a rock-boring bivalve species and associated hard substrate community in fresh water. Our record is of exceptional paleontological, archeological, and environmental importance, because, to our knowledge, until now rock-borers were known only among marine and estuarine groups of macroinvertebrates^8–11,14,15^. From freshwater habitats, only a few records of carbonate microbioerosion structures have been described, i.e., micro-borings in mollusc shells associated with a group of minute shell-boring polychaetes^25,26^. To the best of our knowledge, silicate macrobioerosion in fresh water was previously unknown.

Our findings highlight that rocks with boreholes and fossil members of rock-borer’s assemblages are not necessarily an absolute indicator of shallow marine paleo-environments, but may also reflect past freshwater riverine ecosystems. Additionally, we found that the three most important members of the assemblage, i.e., *L. fluminalis*, *S. deltae*, and *N. gangetica*, are marine-derived, secondary freshwater taxa. This finding is in full agreement with the hypothesis of Annandale^27^, who assumed that *Scaphula* and *Novaculina* are freshwater genera of relict marine origin. In contrast, at least one member of the rock-borer’s assemblage, the sponge *C. ultima*, is an entirely freshwater species that is not known from brackish water bodies^28^. The freshwater fauna of the lower and middle reaches of the Kaladan and Lemro River systems appears to be rather a derivative of the Indian fauna (e.g., indicator taxa such as *N. gangetica*^29,30^, *Lamellidens* aff. *marginalis*^31,32^, and *C. ultima*^28^), but the lack of comparable molecular data from India precludes any final biogeographic conclusion. Our modeling suggests that the shift from an estuarine to a freshwater environment at the rock-borer’s site was a relatively recent event that occurred after the Late Pleistocene. These results support the hypothesis that a few marine or brackish species appear to have the unusual ability to invade freshwater over rapid time scales^33^. However, we show that the invasion of a rock-boring species to a freshwater environment may lead to the origin of an associated hard substrate community representing a mix of primary estuarine and freshwater taxa.

The *Gastrochaenolites* has been considered a marine ichnotaxon produced by endolithic bivalves^8,23^ and, to a lesser degree, by gastropods and sipunculan worms^9^. Our discovery of a freshwater silicate rock-boring organism indicates that fossil macroborings in non-calcareous substrates may have been produced not only by marine invertebrates but also by freshwater lineages of endolithic bivalves. From this perspective, the discovery of fossil pholadid bivalves in Cretaceous Burmese amber may also indicate rather a freshwater paleo-environment than the proximity of resin-producing forests to brackish waters^11^. The ichnospecies *G. anauchen* was described from the Early Pennsylvanian deposits of the USA^24^. Our new record supports the hypothesis of Wilson and Palmer^24^ that the Pennsylvanian borings were formed by endolithic bivalves, because the recent Kaladan borings are morphologically identical to those from the Paleozoic coarse grainstone cobbles. Wilson and Palmer^24^ associated such smooth-sided clavate borings with lithophagid bivalves, but the borings of the pholadid species *L. fluminalis* are also characterized by smooth sides. The empty borings in the freshwater rock-boring site at the Kaladan River are often inhabited by nestling bivalve species, i.e., *S. deltae* and *N. gangetica*. This observation is in agreement with paleontological records that fossil bivalve associations are complicated by frequent records of nestling bivalves, e.g., mytilids and *Hiatella* sp. (Hiatellidae), in the vacated borings^34^.

Human-mediated biological invasions of various species outside their native ranges are a global-scale process^35^, which are accompanied by climatic niche shifts^36^ and rapid evolution of dispersal ability^37^ in invaders. Among the piddocks, alien populations of *Martesia striata* were recorded from the Mediterranean Sea, Hawaii, Islands of the British Isles, and Florida^38^. There is a possibility of further invasion of the freshwater rock-borer’s lineage outside the Kaladan River, because of the Kaladan Multi-Modal Transit Transport Project that is being implemented by the Government of India and the Government of Myanmar. The project involves the development of the transit transport system to Mizoram through Myanmar, with a 158 km inland water transport segment via the Kaladan River^39^, which may facilitate a broad expansion of the rock-borer. The larval development of *L. fluminalis* is unknown, but it is most likely that this species has a planktonic larva as the marine pholadids, e.g., *Barnea* and *Martesia* (Supplementary Note 1). A successful invasion of this freshwater lineage into surrounding larger Asian river basins, including the Ganges, Salween, and Mekong, can lead to unpredictable consequences for native ecosystems that can be comparable to those from other invaders with planktonic larvae^40,41^. In contrast, a freshwater population of the rock-boring mussel is of exceptional importance for scientific research, as it probably represents a lineage with unique physiological adaptations to survive in freshwater environments. Taking into account its possible local range and ancient origin, this lineage should be a focus of special research and conservation efforts.

Our discovery of a freshwater rock-boring assemblage in the Kaladan is in agreement with numerous occurrences of marine-derived groups in rivers of the Oriental Region, which have attracted the attention of scientists for many decades^27,30,42–45^. Fossil freshwater shark assemblages were discovered from the Late Jurassic and Early Cretaceous deposits of the Khorat Plateau in Thailand^46,47^ indicating that the colonization of Asian river systems by marine animals started as early as the mid-Mesozoic. Molecular studies generally support the primary marine origin of most recent marine-derived taxa in tropical fresh waters. There were at least three separate freshwater expansions by stingrays in Southeast Asia, and brackish water habitats may have played an important role for these events as evolutionary bottlenecks^48^. Two freshwater colonization events were recorded in ariid catfishes^49^ and at least one event occurs in Southeast Asian freshwater pufferfishes^50^. The phylogeography of a killifish species, *Aplocheilus panchax*, indicates multiple freshwater colonization events during the Pleistocene^51^. Freshwater prawns of the genus *Macrobrachium* independently colonized fresh waters at least five times^52^ and freshwater polychaetes of the genus *Namanereis* show two distinct invasions^53^.

In summary, Southeast Asia seems to be a long-term arena for wide-scale expansion of marine taxa into fresh water, examples of which are known within different animal families. This pattern may be associated with the presence of a plethora of huge estuarine areas, which may facilitate the adaptation of saltwater organisms into freshwater environments. The land-level change resulting in the combination of progressive tectonic uplift of the mainland^54^ and a gradual decrease of the sea level^55^ may also have contributed to this process through a slow rise of river catchment elevation with a subsequent decrease in the level of tidal saltwater influence in the lower sections of the rivers (Fig. 2). Such a neotectonic evolutionary model seems to be the most appropriate explanation for origin of the freshwater rock-borer’s community in Kaladan River. Several more ancient freshwater colonization events are consistent with the collision of the Asian and Indian plates and the subsequent Tethyan regression, which advanced the cohesive zone shift from shoal sea waters to freshwater environments^44^.

The freshwater lineage of *L. fluminalis* from the Kaladan River is an example of a rock-boring freshwater organism that provides the reliable evidence for freshwater macrobioerosion in silicate rock. Phylogenetically, the rock-boring species is a close relative of the marine piddocks *Barnea* spp. The borings of this species in siltstones belong to the ichnospecies *G. anauchen* which expands the putative range of the saltwater *Gastrochaenolites* into fresh water.

The freshwater rock-borer community in Myanmar includes several nestling macroinvertebrate species, including the arc clam *S. deltae* and the jackknife clam *N. gangetica* which belong to relict marine-derived genera with brackish and freshwater species. Our tectonic modeling indicates that this unusual invertebrate community most likely originated approximately 3.5–14 Kyr ago via the neotectonic uplift of the area leading to a gradual lowering of the sea level and a subsequent shift from an estuarine to a freshwater environment. This example corresponds well to the modern data on multiple and wide-scale expansions of marine taxa into fresh water in Southeast Asia which began as early as the mid-Mesozoic. We assume that such a general biogeographic pattern may be associated with the combination of the progressive tectonic uplift of the mainland and with the subsequent decrease in the level of tidal saltwater influence in the lower sections of the rivers occurring in huge estuarine areas around the mainland. This neotectonic evolutionary model predicts the gradual shift in adaptive zone from shallow marine and brackish waters to freshwater habitats.

Our findings highlight that the rocks with macroborings and fossilized members of rock-boring communities are not a direct indicator of shallow marine paleo-environments, but may also reflect freshwater habitats. A rock-borer lineage from Kaladan River adapted to life in a freshwater environment and having putative planktonic larvae represents a potential international threat in the case it manages to invade the surrounding larger Asian river basins, including the Ganges, Mekong, and Salween Rivers.

## Methods

### Data sampling

The samples were collected from a site in the middle reach of Kaladan River. Several blocks with numerous borings and live representatives of the rock-boring community were obtained from submerged siltstone rocks at depths between 1.0–1.5 m using a large tommy-bar. A water sample was collected with a plastic bottle, filtered (0.45 µm) and stored in the dark before analysis by atomic absorption spectroscopy and ionic chromatography. Macroinvertebrate specimens were collected using forceps and immediately preserved in 96% ethanol. Additionally, four samples of siltstone fragments with rock-boring mussels and accessory taxa were conserved in containers with 96% ethanol.

### Geographic and tectonic modeling

The model of the estuarine and freshwater sections of the Kaladan River was created using ESRI ArcGIS 10 software (www.esri.com/arcgis). The topographic base of the map was created with Natural Earth Free Vector and Raster Map Data (www.naturalearthdata.com), Vector Map (VMap) Level 0 (http://earth-info.nga.mil/publications/vmap0.html), and ASTER GDEM (https://lpdaac.usgs.gov/node/1079). The water levels were obtained from a topographic map (scale 1:500,000; The General Staff of the USSR, map nos. F46–3 and F46–4). The water level at the rock-borer’s site was estimated using linear interpolation. We calculated the length of brackish section of the river based on the maximum tide height that was obtained from the Joint Archive for Sea Level^56^. The land-level change rate (i.e. uplift and sea-level change rate) is needed to reconstruct neotectonic evolution of the lower course of the Kaladan River during the Late Holocene. Unfortunately, data for this part of Myanmar are extremely scarce and uneven. South of our research location, Than Tin Aung et al.^57,58^ and Wang et al.^59^ have attempted to obtain such rates on the basis of field surveys of uplifted sea-level indicators. Their observations pointed out that the long-term land-level change rates in this area contain significant spatial variations. For example, late Holocene average uplift rates at southwestern Cheduba Island can be as high as 3.5–5.2 mm/year, whereas the land-level change rate landward along the eastern coast of Ramree Island may be less than 0.5 mm/year^59^. These two islands are located further south of the Kaladan River, but within a ~500-km-long seismic patch that includes the downstream section of the Kaladan River^60^. Closer to the Kaladan River, Than Tin Aung et al.^58^ also reports similar variations just south of the town of Sittwe, where the land-level change rate changes from more than 4.5 mm/year at the seaward sites to ~2 mm/year at the landward sites. Since the Kaladan River is located near the landward sites in these studies, we conservatively applied a long-term land-level change rate *R* of 0.5–2 mm/year in our tectonic modeling.

###  Mineralogical analyses of rock substrate

The grain size analysis was carried out on an Analysette 22 MicroTec Plus Laser Particle Sizer (Fritsch GmbH—Milling and Sizing, Germany) using a rock sample dispersed in distilled water. An XRD approach was applied to estimate the mineral composition of the substrate using a DRON-3M diffractometer (Bourevestnik Inc., Russia). The BSE images were obtained on an electronic scanning microscope Jeol JSM-6480LV (Jeol Ltd., Japan) with energy-dispersive Oxford X-Max^N^ Silicon Drift Detector and crystal-diffractive INCA Wave-500 WDS spectrometer (Oxford Instrument Ltd., UK). The chemical composition of the rock samples was determined by an XRF analysis using a wavelength-dispersive XRF spectrometer PW 2400 (Philips Analytical, the Netherlands). The microindentation hardness (Vickers test) of the rocks was measured using a PMT-3M Vickers Microhardness Tester (LOMO, Russia) with 100 g load. The tester was calibrated using NaCl crystal with 10 g load. Several fragments of the substrate were placed into briquette and were fixed with epoxy glue with subsequent polishing of the surface. Five indentations were performed on each rock fragment, and both diagonals of indentation mark were measured. A mean microhardness value was calculated based on 20 measurements.

### Ichnological identification of borings

The bivalve borings were identified based on morphological patterns and the type of substrate using the appropriate ichnotaxonomic works^23,24^. Longitudinal cross-sections of the borings were used for morphological investigation.

### Morphological identification of invertebrates

For the morphological study of invertebrate specimens, we used a stereomicroscope (Leica M165C, Leica Microsystems, Germany). The comparative analysis of bivalve taxa was carried out according to the shell shape, structure of the hinge, muscle attachment scars, and umbo position. A gastropod species was identified based on shell shape and marking patterns. The polychaete taxa were identified in accordance with morphology of the external body, everted pharynx, and structures of the parapodia and chaetae. Identification of the sponge species was carried out through analysis of skeleton elements using a light microscope (Olympus CX21, Olympus Corporation, Japan) and scanning electron microscope, SEM (Tescan Vega TS5130MM, Tescan Orsay Holding, Czech Republic). Spicules of each type were purified with potassium dichromate solution (2.5% CrO_3_ in 50% H_2_S), washed and mounted on a slide and specimen stub according to standard methods^61^. Measurements (*n* = 25) were performed under light microscope using an ocular micrometer; spine size was measured under SEM; spicule size is presented as minimum-mean-maximum dimensions.

### DNA extraction and molecular analyses

New sequences were obtained from 24 invertebrate specimens belonging to seven species that were collected from the Kaladan and Lemro rivers, western Myanmar (Table 1). Total genomic DNA was extracted from 96% ethanol-preserved tissue samples using the NucleoSpin^®^ Tissue Kit (Macherey-Nagel GmbH & Co. KG, Germany), according to the manufacturer’s protocol. For molecular analyses, we obtained partial sequences of the following markers: the mitochondrial *cytochrome c oxidase subunit I gene* (*COI*) and *16S ribosomal RNA* (*16S rRNA*), and the nuclear *28S ribosomal RNA* (*28S rRNA*). We were unable to obtain the *28S rRNA* gene sequences from the rock-boring species, and we therefore sequenced another nuclear marker, i.e., the *18S ribosomal RNA* (*18S rRNA*). Additionally, sequencing of the *16S rRNA* gene from samples of *L. fluminalis* and *S. deltae* was also unsuccessful (Table 1).

PCR primers are shown in Supplementary Table 5. We applied four marker-specific PCR algorithms as follows: (i) *COI*: 95 °C (4 min), 94 °C (35–37 repeats, 50 s), 50 °C (50 s), 72 °C (50 s), and 72 °C (5 min); (ii) *16S rRNA*: 95 °C (4 min), 94 °C (35–37 repeats, 50 s), 50 °C (50 s), 72 °C (50 s), and 72 °C (5 min); (iii) *28S rRNA*: 95 °C (4 min), 94 °C (36 repeats, 50 s), 62 °C (50 s), 72 °C (50 s), and 72 °C (5 min); and (iv) *18S rRNA*: 95 °C (4 min), 94 °C (32 repeats, 50 s), 58 °C (50 s), 72 °C (50 s), and 72 °C (5 min). Forward and reverse reactions were executed on an ABI PRISM^®^ 3730 DNA analyzer (Thermo Fisher Scientific Inc., Waltham, MA, USA) with the ABI PRISM^®^ BigDye™ Terminator v. 3.1 reagents kit. The sequences were inspected visually with BioEdit v. 7.2.5^62^.

### Searching for the nearest neighbors

Each molecular sequence was checked via the basic local alignment search tool (BLAST; blast.ncbi.nlm.nih.gov) to search for the most similar sequences in NCBI GenBank. Additionally, we used the nearest-neighbor search algorithm implemented in the Barcoding of Life Data System (BOLD; www.boldsystems.org) with *COI* gene sequences of our specimens.

### Sequence alignment

To estimate the phylogenetic position of three bivalve species, i.e., *L. fluminalis*, *S. deltae*, and *N. gangetica*, we sampled three sequence data sets, namely, “Pholadidae & relatives” (two partitions: *18S rRNA* *+* *28S rRNA*), “Arcidae & Noetiidae” (two partitions: *28S rRNA* *+* *18S rRNA*), and “Pharidae” (five partitions: 3 codons of *COI* + *16S rRNA* + *28S rRNA*), respectively (see Supplementary Table 6 for additional sequences obtained from GenBank). Sequences of the marine clam *Cavatidens omissa* Iredale, 1930 (Lucinidae) were used as an outgroup (Supplementary Table 6). The number of partitions in each data set was selected in accordance with the presence of available sequences for most closely related taxa in GenBank. The multiple sequence alignment was produced for each gene in each data set separately in MEGA6^63^, with the Muscle (“Arcidae & Noetiidae” and “Pholadidae & relatives” data sets) and ClustalW (“Pharidae” data set) algorithms. The aligned sequence data sets were inspected with GBlocks v. 0.91b (Supplementary Table 7) to exclude gaps and variable sections from the alignments using a less strict set of options. Lacking sites were coded as missing positions.

### Saturation analyses and congruence of phylogenetic signals

To estimate each partition in each data set for evidence of substitution saturation, we used the test of Xia et al. with DAMBE v. 5.3.108^64^. The test revealed very little saturation even under the hypothesis of an asymmetrical tree. A partition homogeneity test was calculated in PAUP* v. 4.0a150 to check the congruence of phylogenetic signals among sequence partitions^65^. This test returned the conformity of the phylogenetic signals among the partitions in the “Arcidae & Noetiidae” data set (*P* = 0.99). For the “Pharidae” data set, signals were also congruent (*P* > 0.3), with exception of those between the *COI* and *28S rRNA* genes (*P* = 0.01) although there was no significant discordance between the *16S rRNA* and *28S rRNA* genes (*P* = 0.34). We concluded that the incongruence between these data partitions does not affect the model because it appears to be the effect of copious homoplasy^31^. We therefore used the combined data set (3 codons of *COI* + *16S rRNA* + *28S rRNA*) in phylogenetic analyses. In contrast, the test indicated significant incongruence between the *18S* and *28S rRNA* genes in the “Pholadidae & relatives” data set (*P* = 0.01). However, testing of a restricted data set, which includes only the members of this family and an outgroup taxon, returned no conflicts among partitions (*P* = 1.00). We therefore used this “Pholadidae” data set in subsequent reconstructions.

### Phylogenetic analyses

Phylogenetic analyses were carried out with RAxML v. 8.2.6 HPC Black Box^66^ and MrBayes v. 3.2.6^67^ at the San Diego Supercomputer Center through the CIPRES Science Gateway^68^. In the RAxML analyses, a GTR+G model was selected for each partition. Nodal support values were calculated with a standard bootstrapping approach^66^. The evolutionary models for each partition according to the corrected Akaike Information Criterion (AICc) of MEGA6^63^ that were used under Bayesian inference framework are presented in Supplementary Table 8. The MrBayes analyses were carried out for 25,000,000 generations with the following options: two runs, four Markov chains (three cold and one heated with temperature of 0.1), and sampling every 1000th cycle; 10% of the samples were excluded as an appropriate burn-in, and the convergence of the Markov chains was inspected with Tracer v. 1.6^69^.

### Ancestral area reconstructions

To estimate the ancestral area patterns, we calculated the sets of uncalibrated phylogenetic trees for each data set, i.e., “Pholadidae”, “Arcidae & Noetiidae”, and “Pharidae”, using BEAST 2 v. 2.4.6 with a lognormal relaxed clock algorithm and the Yule speciation process as the tree prior^70^. Models were calculated at the San Diego Supercomputer Center through the CIPRES Science Gateway^68^. We applied the same options to the data sets as in the MrBayes analyses (see above). However, we selected simplified substitution models (see Supplementary Table 8). In each case, two independent runs with 25,000,000 generations were carried out. The phylogenies were reconstructed every 1000th cycle. The primary log data sets were inspected visually with Tracer v. 1.6 for the congruence of the Markov chains and the effective sample size of parameters (ESS)^69^. The ESS values for all parameters were found as >350, and the posterior and prior distributions were congruent. For each data set, the phylogenies from two separate runs were joined with LogCombiner v. 1.8.3 (burn-in = 10%), and the maximum clade credibility tree was reconstructed with TreeAnnotator v. 1.8.3^71^.

For each sequence data set, a sample of post-burn-in binary trees (*N* = 45,002) and a user-specified consensus tree obtained from BEAST runs were used to calculate ancestral area patterns on the basis of three algorithms, i.e., Statistical Dispersal-Vicariance Analysis (S-DIVA), Dispersal-Extinction Cladogenesis (DEC), and Statistical Dispersal-Extinction Cladogenesis (S-DEC) implemented in RASP v. 3.2^72^. The out-group taxa were discarded from the phylogenies with RASP v. 3.2. Additionally, only one representative of each species was used in RASP analyses to avoid uncertainty of the biogeographic models. We assigned two possible ancestral areas of the in-group species, i.e., (a) marine and (ab) estuarine to freshwater. The S-DIVA scenarios were computed with two allowed areas, allowing 100 reconstructions with maximum reconstruction for final tree = 1000, and allowing extinctions. The DEC and S-DEC scenarios were reconstructed with default options and two allowed areas. Additionally, we calculated a combined model (S-DEC + DEC + S-DIVA) joining the independent scenarios inferred from three different algorithms in RASP v. 3.2.

### Data availability

The sequences generated under this study are available from GenBank. Accession numbers for each specimen are presented in Table 1. The voucher specimens and samples of siltstone blocks with boreholes are available in the RMBH, Russian Museum of Biodiversity Hotspots, the Federal Center for Integrated Arctic Research, Russian Academy of Sciences (Arkhangelsk, Russia).

## Electronic supplementary material

Supplementary Information
