# Supplementary material for: Discovery of a silicate rock-boring organism and macrobioerosion in fresh water
Source: Nat Commun. 2018 Jul 23;9:2882. doi: 10.1038/s41467-018-05133-4 (PMC6056532; doi:10.1038/s41467-018-05133-4)
Supplement: Supplementary file 1 — Supplementary Information [file 41467_2018_5133_MOESM1_ESM.pdf]

SUPPLEMENTARY INFORMATION

Discovery of a silicate rock-boring organism and macrobioerosion in fresh water

Bolotov *et al.*

Contents

Supplementary Table 1. Hydrochemical characteristics of a water sample (no. 114W*) from the freshwater bioerosion site at the Kaladan River, western Myanmar.

Supplementary Table 2. Mineral composition (%) of the rock substrate of the rock-boring bivalves from the Kaladan River.

Supplementary Table 3. Chemical composition (%) of the rock substrate of the rock-boring bivalves from the Kaladan River.

Supplementary Table 4. Nearest neighbors of freshwater rock-borer community's members from Kaladan River, western Myanmar on the basis of BOLD IDS and of BLAST search via GenBank (accessed 03.09.2017).

Supplementary Table 5. Primer sequences for PCR amplification and sequencing.

Supplementary Table 6. List of additional sequences used in this study for phylogenetic reconstructions.

Supplementary Table 7. Alignment length prior to and after treatment in GBlocks v. 0.91b.

Supplementary Table 8. Models of sequence evolution for each partition based on corrected Akaike Information Criterion (AICc) of MEGA6 that were applied within Bayesian models.

Supplementary Fig. 1. Backscattered electron (BSE) images and grain size curve of rock substrate of the rock-boring bivalves from the Kaladan River. (**a-c**) BSE images of polished surface of (**a**) sample no. 1, (**b**) sample no. 2, and (**c**) sample no. 3. (**d**) Grain size curve (sample no. 1).

Supplementary Figure 2. Ultrametric Bayesian (BEAST 2) phylogeny showing the primary marine origin of *Scaphula deltae* (*28S rRNA* + *18S rRNA*, 2450 bp), a nestling bivalve species from the bioerosion site in the Kaladan River, western Myanmar. Black numbers near nodes are BPP values inferred from BEAST. Pie chaps on the nodes indicate the probabilities of certain ancestral areas with respect to combined results under three different models (S-DIVA, DEC and S-DEC) inferred from RASP v. 3.2. An outgroup taxon (*Cavatidens omissa*) is not shown.

Supplementary Figure 3. Morphological features of *Corvospongilla ultima*, a nestling freshwater sponge species from the bioerosion site in the Kaladan River, western Myanmar. (**a**) Skeleton of dry sponge (scale bar = 300 μm). (**b**) Skeletal mesh (Scale bar = 100 μm). (**c**) Spicule composition, including (c1) megascleres, (c2) details of megasclere surface, (c3) microslere, (c4) gemmuloscleres, and (c5) strongyles of gemmular cage. (Photos: Agniya M. Sokolova).

Supplementary Note 1. Taxonomic Account.

Supplementary Table 1. Hydrochemical characteristics of water sample no. 114W* from the freshwater bioerosion site at the Kaladan River, western Myanmar

**Macro-ions**	K^+	Na^+	Mg^{2+}	Ca^{2+}	F^-	Cl^-	SO_4^{2-}
Concentration (mean ± SD), mg/L	2.22±0.33	11.6±1.7	7.84±0.78	15.5±1.6	0.11±0.02	2.67±0.40	9.62±1.44

*This sample was collected 01.v.2015 (dry season). Salinity value was 0.16‰.

Supplementary Table 2. Mineral composition (%) of the rock substrate of the rock-boring bivalves from the Kaladan River

**Mineral**	**Sample no. 1 (S-1)**	**Sample no. 3 (S-3)**
Smectite	8	13
Illite	24	23
Chlorite	7	9
Kaolinite	15	10
Quartz	29	30
K-feldspar	3	<1
Plagioclase	12	7
Pyroxene	<1	1
Calcite	<1	2
Siderite	<1	1
Goethite	<1	2
Pyrite	2	2

Supplementary Table 3. Chemical composition (%) of the rock substrate of the rock-boring bivalves from the Kaladan River

**Component**	**Sample no. 1**	**Sample no. 2**	**Sample no. 3**	**Mean ± SD**
Na_2O	1.02	1.07	1.12	1.07±0.05
MgO	2.25	2.29	2.24	2.26±0.03
Al_2O_3	16.76	17.42	16.69	16.96±0.40
SiO_2	57.11	56.38	57.30	56.93±0.49
P_2O_5	0.21	0.13	0.09	0.14±0.06
SO_3*	0.53	0.15	0.22	0.30±0.20
K_2O	3.30	3.54	3.43	3.42±0.12
CaO	0.80	0.75	0.84	0.80±0.05
TiO_2	0.87	0.87	0.89	0.88±0.01
MnO	0.10	0.14	0.10	0.11±0.02
FeO**	6.32	6.13	5.80	6.08±0.26
Total	89.28	88.83	88.69	88.93±0.31

*Total S is calculated as SO_3. **Total Fe is calculated as FeO.

Supplementary Table 4. Nearest neighbors of freshwater rock-borer community's members from Kaladan River, western Myanmar on the basis of BOLD IDS and of BLAST search via GenBank (accessed 03.ix.2017)

**Family: Species**	**Marker**	**Nearest neighbors on the basis of BOLD IDS (N is a number of sequences)**	**Similarity, %**	**Nearest neighbors on the basis of BLAST search via GenBank**	**Similarity, %**
Pholadidae: *Lignopholas fluminalis* (Blanford, 1867)	*COI*	Pholadidae: *Barnea davidi* (Deshayes, 1874) (*N* = 2)	81.41	Pholadidae: *Barnea* spp.	79-82
	18S rRNA	n/a	n/a	Pholadidae: *Barnea parva* (Pennant, 1777), *B. candida* (L., 1758), *B. truncata* (Say, 1822)	98-99
Arcidae: *Scaphula deltae* Blanford 1867	*COI*	Noetiidae: *Noetia ponderosa* (Say, 1822) (*N* = 6)	77-18-77.64	n/a	n/a
	28S rRNA	n/a	n/a	Unresolved (several genera in different families)	91-92
Pharidae: *Novaculina gangetica* Benson, 1830	*COI*	Pharidae: *Sinonovacula constricta* (Lamarck, 1818) (*N* = 99)	87.06-88.04	Pharidae: *Sinonovacula constricta* (Lam., 1818)	87.39
	16S rRNA	n/a	n/a	Pharidae: *Sinonovacula rivularis* Huang et Zhang, 2007	92.40
	28S rRNA	n/a	n/a	Pharidae: *Sinonovacula constricta* (Lamarck, 1818)	90.84
Neritidae: *Clithon* cf. *reticularis* (Sowerby, 1838)	*COI*	Neritidae: *Clithon spinosum* (G. B. Sowerby I, 1825) (*N* = 28)	90.83-91.67	Neritidae: *Clithon lentiginosum* (Reeve, 1855)	91
	16S rRNA	n/a	n/a	Neritidae: *Clithon retropictum* (Martens, 1879)	96
	28S rRNA	n/a	n/a	Neritidae: *Neritina virginea* (L., 1758)	100
Nereididae: *Neanthes meggitti* (Monro, 1931)	*COI*	Nereididae: *Pseudonereis gallapagensis* Kinberg, 1865 (*N* = 3)	82.04-82.44	n/a	n/a
	16S rRNA	n/a	n/a	Nereididae: *Hediste atoka* Sato et Nakashima, 2003	86
	28S rRNA	n/a	n/a	Nereididae: *Nereis pelagica* L., 1758	90
Nereididae: *Namalycastis indica* (Southern, 1921)	*COI*	Nereididae: *Namalycastis* spp. (*N* = 28)	80.43-81.86	Polychaeta sp. EBS12u-Po1	88
	16S rRNA	n/a	n/a	Nereididae: *Namalycastis abiuma* (Grube, 1872)	84
	28S rRNA	n/a	n/a	n/a	n/a
Spongillidae: *Corvospongilla ultima* (Annandale, 1910)	*COI*	Lubomirskiidae: *Baikalospongia intermedia* Dybowsky, 1880 (*N* = 1); Spongillidae: *Spongilla lacustris* (L., 1759) (*N* = 13)	98.48-99.39	Spongillidae: *Spongilla lacustris* (L., 1759)	99
	28S rRNA	n/a	n/a	Spongillidae: *Eunapius* sp. NHMUK 2009.12.22.17	92

Supplementary Table 5. Primer sequences for PCR amplification and sequencing

Gene fragment	Primer's name	Direction	Sequence (5′-3′)	Reference
COI	LCO1490	Forward	ggtcaacaaatcataaagatattgg	Ref. 1
	HCO2198	Reverse	taaacttcagggtgaccaaaaaatca	
	LoboF1	Forward	kbtchacaaaycayaargayathgg	Ref. 2
	LoboR1	Reverse	taaacytcwggrtgwccraaraayca	
16S rRNA	16Sar	Forward	cgcctgtttatcaaaaacat	Ref. 3
	16sar-L-myt	Forward	cgactgtttaacaaaaacat	Ref. 4
	16sbr-H-myt	Reverse	ccgttctgaactcagctcatgt	
28S rRNA	C1	Forward	acccgctgaatttaagcat	Ref. 5
	D2	Reverse	tccgtgtttcaagacgg	
18S rRNA	1F	Forward	tacctggttgatcctgccagtag	Ref. 6
	4R	Reverse	gaattaccgcggctgctgg	
	3F	Forward	gttcgattccggagaggga	
	18Sbi	Reverse	gagtctcgttcgttatcgga	Ref. 7
	18Sa2.0	Forward	atggttgcaaagctgaaac	
	9R	Reverse	gatccttccgcaggttcacctac	Ref. 5

Supplementary Table 6. List of additional sequences from GenBank used in this study for phylogenetic reconstructions

Taxa*	Environment*	Locality	*COI*	*16S rRNA*	*28S rRNA*	*18S rRNA*
**Pholadidae**						
Barnea candida (L., 1758)	Marine	Wachapreague, VA			AM779715	AM774541
Barnea parva (Pennant, 1777)	Marine	Sussex, UK			AM779716	AM774542
Barnea truncata (Say, 1822)	Marine	Wachapreague, VA			JF899178	JF899206
Martesia striata (L., 1758)	Marine to brackish	Minahasa, Indonesia			JF899185	JF899213
Pholas dactylus L., 1758	Marine	Charmouth, Dorset, UK			JF899192	JF899220
Cyrtopleura costata (L., 1758)	Marine	Wachapreague, VA			JF899179	JF899207
**Teredinidae**						
Bankia australis (Calman, 1920)	Marine	Manado Bay, Indonesia			JF899174	JF899202
B. carinata (J.E. Gray, 1827)	Marine	Lac Bay, Bonaire, Netherlands Antilles			JF899175	JF899203
B. gouldi (Bartsch, 1908)	Marine	Newport River, Beaufort, NC			JF899176	JF899204
B. setacea (Tryon, 1863)	Marine	Brown's Bay, WA			JF899177	JF899205
Dicyathifer mannii (Wright, 1866)	Marine	Minahasa Peninsula, Indonesia			JF899180	JF899208
Kuphus polythalamia (L., 1758)	Marine	Zamboanga del Sur, Mindanao, Philippines			JF899182	JF899210
Lyrodus massa (Lamy, 1923)	Marine	Manado Bay, Indonesia			JF899183	JF899212
L. pedicellatus (Quatrefages, 1849)	Marine	Banana River, FL, mangrove wood			JF899184	JF899211
Nausitora dunlopei Wright, 1864	Marine to brackish	Minahasa Peninsula, Indonesia			JF899187	JF899215
N. fusticulus (Jeffreys, 1860)	Marine	Praia Dura, Ubatuba, Brazil			JF899188	AY192697
Neoteredo reynei (Bartsch, 1920)	Marine	Praia Dura, Ubatuba, Brazil			JF899189	JF899217
Spathoteredo obtusa (Sivickis, 1928)	Marine	Manado Bay, Indonesia			JF899193	JF899221

Taxa*	Environment*	Locality	*COI*	*16S rRNA*	*28S rRNA*	*18S rRNA*
Teredo navalis L., 1758	Marine to brackish	Collection panels, Belfast pier, Belfast, ME			JF899194	JF899222
Teredora malleolus (Turton, 1822)	Marine	Lagoen, Bonaire, NA, driftwood			JF899195	JF899223
Teredothyra dominicensis (Bartsch, 1921)	Marine	Bachelor's Beach, Bonaire, NA, 3 m			JF899197	JF899225
**Xylophagaidae****						
Xylophaga atlantica Richards, 1942	Marine	12 miles east of Southwest Harbor, ME, 100 m			AY070132	AY070123
X. sp.	Marine	SE of Port Dunford (29°02.20S, 32°19.60E), 800 m			JF899198	JF899226
X. washingtona Bartsch, 1921	Marine	Friday Harbor, WA			JF899199	JF899227
Xylopholas sp.	Marine	Gulf of Mexico (27°44.750' N, 91°13.31' W), 540 m			JF899200	JF899228
Xyloredo sp.	Marine	Gulf of Mexico (27°44.750' N, 91°13.31' W), 540 m			JF899201	JF899229
**Pharidae**						
Ensiculus cultellus (L., 1758)	Marine	Queensland, Australia			AM779682	
Ensis leei Huber, 2015	Marine	Europe & USA (chimeric sequence)	EU523673	HF970450	JF909603	
Ensis siliqua (L., 1758)	Marine	Europe (chimeric sequence)	EU523683		KX713382	
Pharella javanica (Lam., 1818)	Marine	Thailand			AM779683	
Pharus legumen (L., 1758)	Marine	South Wales, UK			AM779684	
Phaxas pellucidus (Pennant, 1777)	Marine	Sweden, Tjärnö	KC429145	KC429309	KC429508	
Siliqua alta (Broderip et Sowerby, 1829)	Marine	Hokkaido, Japan		AB751362	AB746908	
Sinonovacula constricta (Lam., 1818)	Marine	China	JN859986	JN859901	AF131005	
S. rivularis Huang et Zhang, 2007	Marine	China		EU169036		
**Noetiidae*****						
Arcopsis adamsi (Dall, 1886)	Marine	Florida, USA			KC429419	KC429327
Arcopsis sp.	Marine	Guangxi, China			JN974568	JN974519
Didimacar tenebrica (Reeve, 1844)	Marine	Zhejiang, China			JN974566	JN974516
Noetia ponderosa (Say, 1822)	Marine	South Carolina, USA			KT757840	KT757793
Striarca lactea (L., 1758)	Marine	Blanes, Spain			KT757855	AF120531
S. lactea (L., 1758)	Marine	Roses, Spain			KT757856	KT757809
Verilarca interplicata (Grabau & King, 1928)	Marine	Shandong, China			JN974569	JN974520
V. interplicata (Grabau & King, 1928)	Marine	Shandong, China			JN974570	JN974521
**Arcidae*****						
Acar bailyi Bartsch, 1931	Marine	Baja California, Mexico			KT757813	KT757765
A. domingensis (Lamarck, 1819)	Marine	Bahamas			KT757814	KT757766
Arca imbricata Bruguière, 1789	Marine	Florida, USA			KT757818	KT757771

Taxa*	Environment*	Locality	*COI*	*16S rRNA*	*28S rRNA*	*18S rRNA*
A. navicularis Bruguière, 1789	Marine	Queensland, Australia			KT757821	KT757774
A. noae L., 1758	Marine	Roses, Spain			KT757822	KT757775
A. patriarchalis Röding, 1798	Marine	Guangxi, China			JN974576	JN974527
A. boucardi Jousseaume, 1894	Marine	Shandong, China			JN974577	JN974529
A. zebra (Swainson, 1833)	Marine	Bocas, Panama			KT757823	KT757776
Barbatia amygdalumtostum (Röding, 1798)	Marine	Hainan, China			JN974575	JN974526
B. barbata (L., 1758)	Marine	Blanes, Spain			KC429417	KC429326
B. foliata (Forsskål in Niebuhr, 1775)	Marine	Guangxi, China			JN974562	JN974511
B. lacerata (Bruguière, 1789)	Marine	Guangxi, China			JN974560	JN974509
B. sp.	Marine	Queensland, Australia			KT757832	KT757785
B. virescens (Reeve, 1844)	Marine	Zhejiang, China			JN974573	JN974524
Bathyarca glomerula (Dall, 1881)	Marine	Colombia (Caribbean)			KT757837	KT757790
Trisidos kiyonoi (Makiyama, 1931)	Marine	Hainan, China			JN974571	JN974522
T. tortuosa (L., 1758)	Marine	Queensland, Australia			KT757858	KT757811
Anadara antiquata (L., 1758)	Marine	Hainan, China			JN974542	JN974491
A. broughtonii (Schrenck, 1867)	Marine	Jiangsu, China			JN974550	JN974499
A. cornea (Reeve, 1844)	Marine	Hainan, China			JN974546	JN974495
A. crebricostata (Reeve, 1844)	Marine	Guangxi, China			JN974540	JN974489
A. globosa (Reeve, 1844)	Marine	Hainan, China			JN974534	JN974484
A. gubernaculum (Reeve, 1844)	Marine	Hainan, China			JN974544	JN974493
A. inaequivalvis (Bruguière, 1789)	Marine	Hainan, China			JN974548	JN974497
A. notabilis (Röding, 1798)	Marine	Florida, USA			KT757816	KT757768
A. pilula (Reeve, 1843)	Marine	Hainan, China			JN974558	JN974507
A. sativa (Bernard et al., 1993)	Marine	Guangxi, China			JN974552	JN974501
A. trapezia (Deshayes, 1839)	Marine	Queensland, Australia			KT757817	KT757770
A. vellicata (Reeve, 1844)	Marine	Guangxi, China			JN974538	JN974487
Tegillarca granosa (L., 1758)	Marine	Hainan, China			JN974556	JN974505
T. nodifera (Martens, 1860)	Marine	Jiangsu, China			JN974554	JN974503
**Lucinidae**						
Cavatidens omissa Iredale, 1930****	Marine	Moreton Bay, Australia	KC429120	KC429277	KC429465	KC429363

*Taxonomy and environmental preference are on the basis of the World Register of Marine Species database[8]. **New spelling of the family name in accordance with Coan et al.[9]. ***Species names are in accordance with Combosch and Giribet[10]. ****Outgroup taxon.

Supplementary Table 7. Alignment length prior to and after treatment in GBlocks v. 0.91b[11]

Data set and partition	Original length of alignment (bp)	Fraction selected by GBlocks (%)	Final length of alignment (bp)
“Pholadidae”			
18S rRNA	2064	83	1733
28S rRNA	3112	41	1300
“Pharidae”			
COI	659	99	658
16S rRNA	526	90	474
28S rRNA	3025	48	1470
“Arcidae & Noetiidae”			
28S rRNA	3114	22	692
18S rRNA	1967	89	1758

Supplementary Table 8. Models of sequence evolution for each partition based on corrected Akaike Information Criterion (AICc) of MEGA6[12] that were applied within Bayesian models

Data set and partition	Model	Gamma	Invariant
“Pholadidae”			
18S rRNA	GTR*+G	0.29	n/a
28S rRNA	TN93+G+I	0.46	0.35
“Pharidae”			
1st codon of *COI*	HKY+G	4.13	n/a
2nd codon of *COI*	TN93+I	n/a	0.45
3rd codon of *COI*	GTR*	n/a	n/a
16S rRNA	GTR*+G	0.18	n/a
28S rRNA	GTR*+G	0.25	n/a
“Arcidae & Noetiidae”			
28S rRNA	GTR*+G+I	0.49	0.26
18S rRNA	GTR*+G+I	0.45	0.54

*We used HKY model instead of the GTR in BEAST analyses (see Bolotov et al.[13] for explanation). n/a – not available.

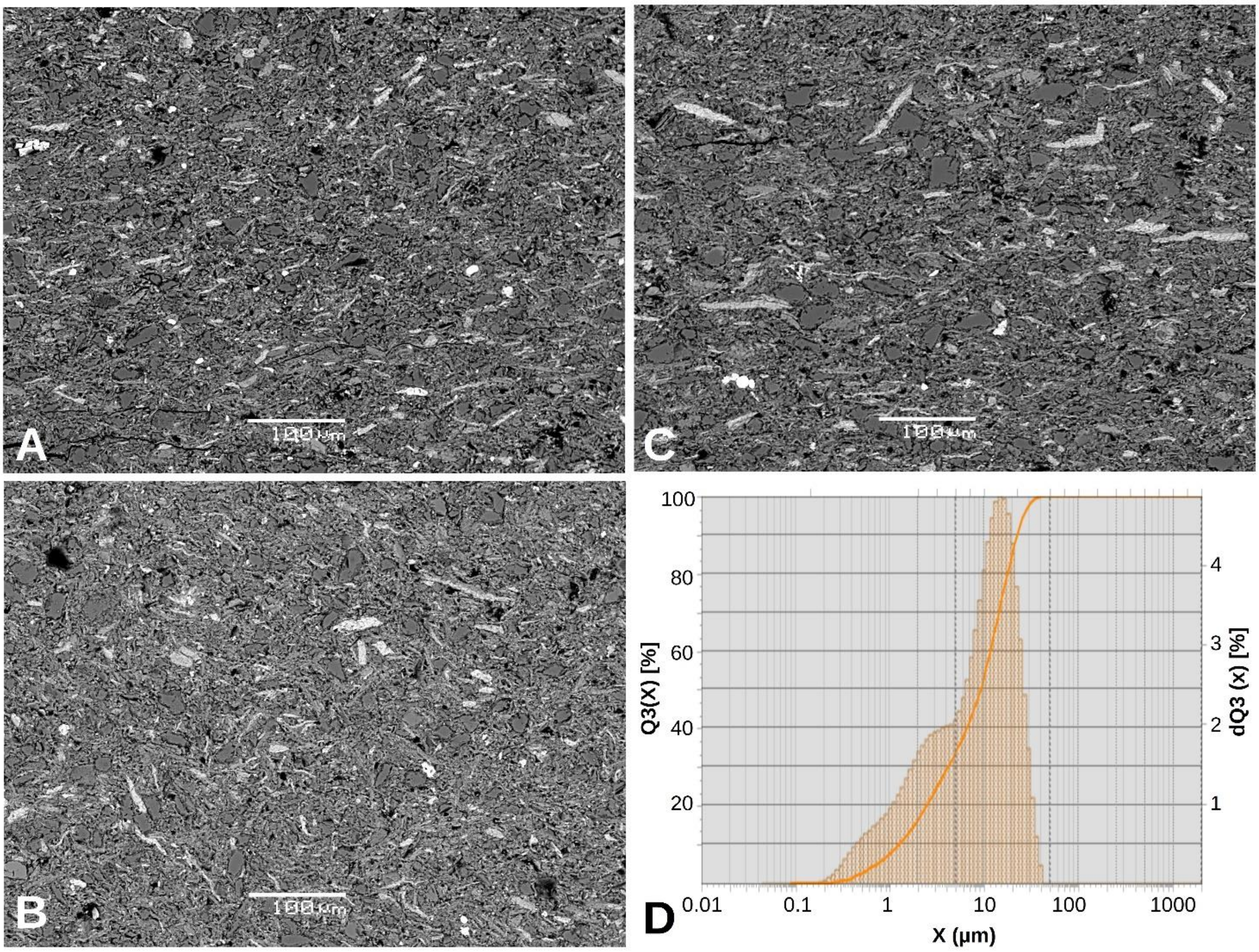

Supplementary Figure 1. Backscattered electron (BSE) images and grain size curve of rock substrate of the rock-boring bivalves from the Kaladan River. (**a-c**) BSE images of polished surface of (**a**) sample no. 1, (**b**) sample no. 2, and (**c**) sample no. 3. (**d**) Grain size curve (sample no. 1).

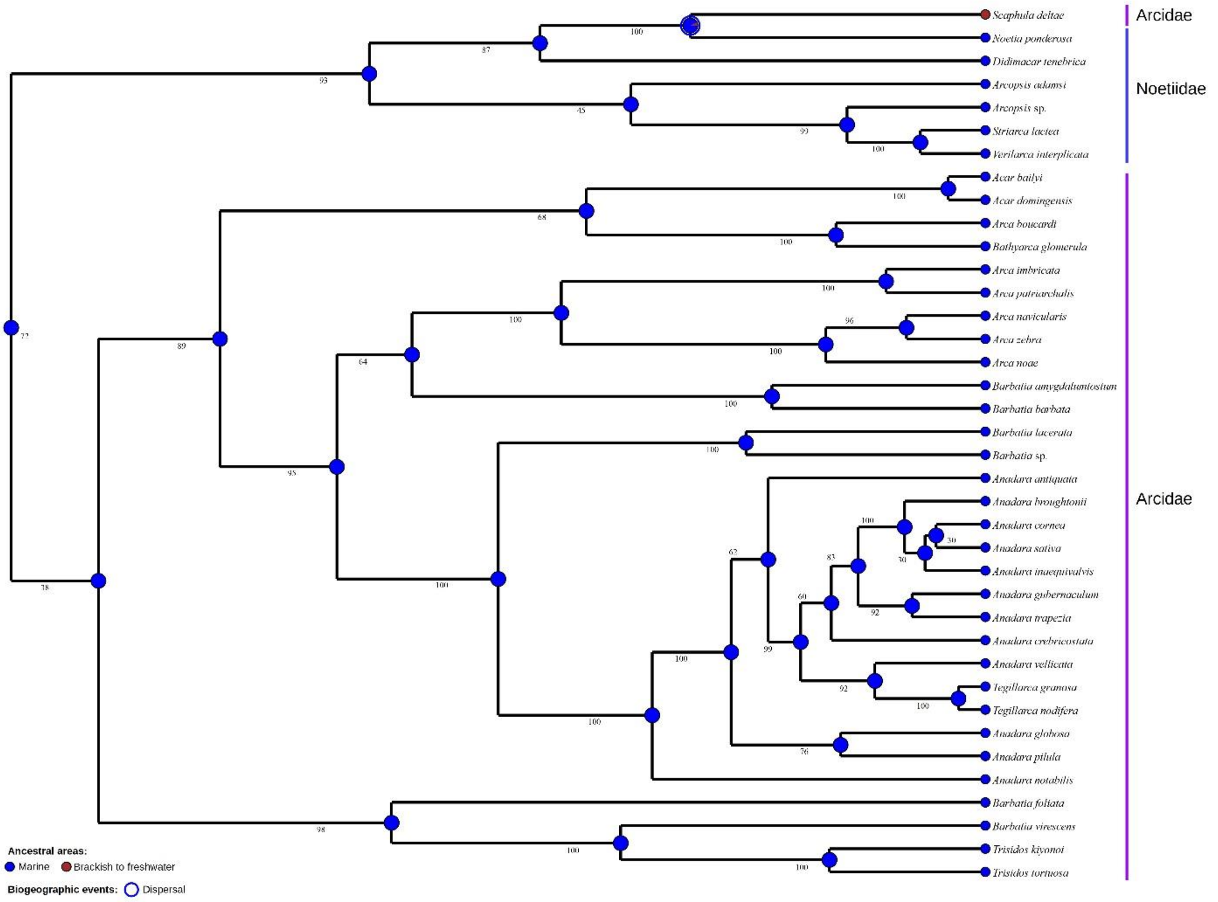

Supplementary Figure 2. Ultrametric Bayesian (BEAST 2) phylogeny showing the primary marine origin of *Scaphula deltae* (*28S rRNA* + *18S rRNA*, 2450 bp), a nestling bivalve species from the bioerosion site in the Kaladan River, western Myanmar. Black numbers near nodes are BPP values inferred from BEAST. Pie chaps on the nodes indicate the probabilities of certain ancestral areas with respect to combined results under three different models (S-DIVA, DEC and S-DEC) inferred from RASP v. 3.2. An outgroup taxon (*Cavatidens omissa*) is not shown.

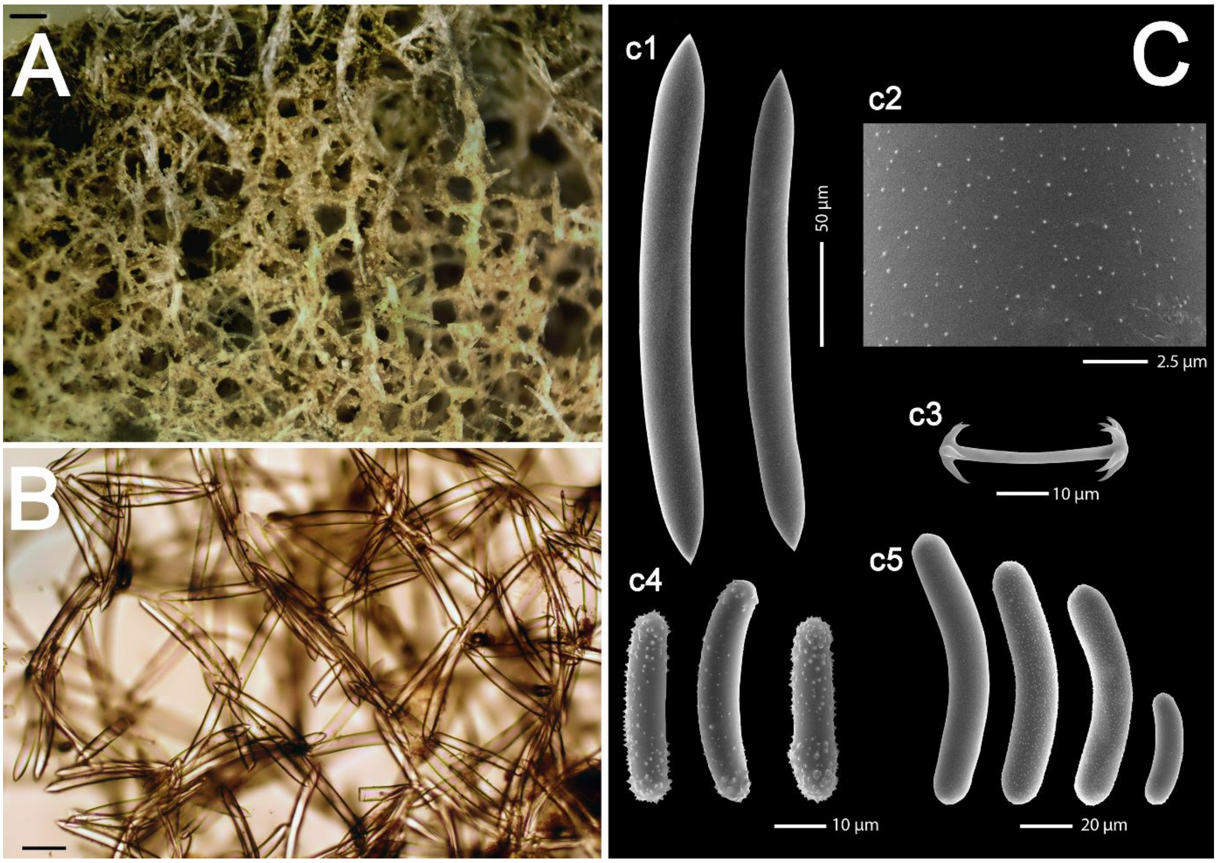

Supplementary Figure 3. Morphological features of *Corvospongilla ultima*, a nestling freshwater sponge species from the silicate bioerosion site in the Kaladan River, western Myanmar. (**a**) Skeleton of dry sponge (scale bar = 300 µm). (**b**) Skeletal mesh (scale bar = 100 µm). (**c**) Spicule composition, including (c1) megascleres, (c2) details of megasclere surface, (c3) microslere, (c4) gemmuloscleres, and (c5) strongyles of gemmular cage (scale bars are given on the image). (Photos: Agniya M. Sokolova).

Supplementary Note 1. Taxonomic Account

Phylum Mollusca Linnaeus, 1758

Class Bivalvia Linnaeus, 1758

Family Pholadidae Lamarck, 1809

Lignopholas Turner, 1955

Type species: *Lignopholas clappi* Turner, 1955 (by original designation)

Lignopholas fluminalis (Blanford, 1867)

Martesia fluminalis Blanford, 1867: 67[14].

Lignopholas fluminalis (Blanford, 1867): Turner & Santhakumaran (1989): 175[15].

Material examined: Myanmar: Kaladan River, 21.0094° N, 92.9813° E, submerged siltstone rocks with boreholes, 01.v.2015, 39 specimens, Bolotov, Vikhrev, Aksenova, Konopleva, & local villagers leg.

Morphology: Our sample is almost identical to the typical *Lignopholas fluminalis*, although some specimens from the Kaladan have more elongated and narrower shell. Shell ovate-conical, thin, especially in anterior area. Anterior part of shell separated from posterior part by oblique line, which runs from dorsal (hinge area) to ventral margin. Anterior part hemispherical, area around upper side covered by closely-spaced lines, dispersed from top; part near ventral margin smooth. Hinges covered by trilobite callum, divided in center. Posterior part acuminated, with concentric lines, and with fringed lamellae along the dorsal margin on each valve. Periostracum white-sandy. There is a myophore in both valves. Adductor scars shallow. Anterior scar invisible. Posterior adductor scar elongate-elliptical, located along dorsal side.

Distribution and ecology: Graf[16] placed *Lignopholas fluminalis* among freshwater bivalves but instead it is *L. rivicola* that should be listed as a freshwater species[15], not *L. fluminalis* (D. Graf, pers. comm., 2018), because the latter species was previously found only in estuarine environments in Myanmar (delta of the Irrawaddy and Pegu rivers), India and Borneo[15]. Kaladan's population of *Lignopholas fluminalis* seems to be the unique lineage adapted to freshwater habitats. The larval development of *Lignopholas fluminalis* is unknown, but it is most likely that this species has a planktonic larva as marine taxa of the Pholadidae, e.g., *Barnea* and *Martesia*[17,18].

Comments: The species differs from taxa of the genus *Lignopholas* Turner, 1955 because it is a silicate rock borer which can bore into siltstone rocks, brickwork and laterites, and at least one lineage of this estuarine species invades freshwater habitats, whereas the other three species in the genus are exclusively brackish woodborers (this study; Ref. 14,15,19). The molecular sequences of the type species of the genus are not available.

Family Arcidae Lamarck, 1809

Genus *Scaphula* Benson, 1834

Type species: *Scaphula celox* Benson, 1836 (by monotypy)

Scaphula deltae Blanford, 1867

Material examined: Myanmar: Kaladan River, 21.0094° N, 92.9813° E, submerged siltstone rocks with boreholes, 01.v.2017, 31 specimens, Bolotov, Vikhrev, Aksenova, Konopleva, & local villagers leg.

Morphology: Our sample differs by broader and more curved posterior margin. Shell small, subtrapezoidal, equivalve, rather thick, inflated. Posterior margin broader than anterior margin, curved. Kiel high and sharp, divided the valves into two parts. Periostracum olivaceous; nacre yellow-whitish. Hinge with numerous small teeth; four converging anterior and six oblique posterior teeth, place between them edentate. Adductor scars somewhat squarish or roundish.

Distribution and ecology: An associated nesting species, specimens of which were byssally attached to submerged siltstone rocks (on the rock surface and in empty borings) in a single site of Kaladan River, western Myanmar. The nominal taxon *Scaphula deltae* Blanford, 1867 was described from the delta of Irrawaddy[14] but it was also recorded from India and Bangladesh, including the freshwater section of Ganges River in 1,500 km upstream from the delta[19-22]. Annandale[19] found this species in empty borings of *Lignopholas fluminalis* that were situated in a submerged brickwork in a brackish section of the Ganges River.

Comments: The genus *Scaphula* was considered a member of the Arcidae but it clusters within the Noetiidae Stewart, 1930, with *Noetia ponderosa* (Say, 1822) as the nearest neighbor of *S. deltae* (Fig. 5, Supplementary Fig. 2 and Supplementary Table 4). However, we cannot transfer this genus to the latter family, because the sequences of other *Scaphula* taxa, including the type species, are not available. Additionally, phylogenetic relationships among Arcoidea families are still unresolved, with the family Noetiidae appearing as a subgroup within, rather than as a sister group to, the Arcidae[10,23]. The genus *Scaphula* represents a small, distinct group of morphologically similar species with restricted distribution range in India, Myanmar, Bangladesh, Thailand and Vietnam[21,22,24], which appear to be phylogenetically close to each other.

Family Pharidae H. Adams & A. Adams, 1856

Genus *Novaculina* Benson, 1830

Type species: *Novaculina gangetica* Benson, 1830 (by monotypy)

Novaculina gangetica Benson, 1830

Material examined: Myanmar: Kaladan River, 21.0094° N, 92.9813° E, submerged siltstone rocks with boreholes, 01.v.2015, 4 specimens, Bolotov, Vikhrev, Aksenova, Konopleva, & local villagers leg.; Lemro River, 20.6150° N, 93.2481° E, gravel-clay bottom, 30.iv.2015, 35 specimens, Bolotov, Vikhrev, Aksenova, Konopleva, & local villagers leg.

Morphology: Our sample is similar to specimens from the Ganges but differs by the less prominent umbo. Shell oblong, rather thin, inequilateral, not inflated, with truncated or rounded dorsal margin. Periostracum olivaceous, with dark bands; nacre white-bluish, shining. Umbo not prominent, corrugated; beak sculpture not strong. There are two small pseudocardinal teeth on right valve and three small pseudocardinal teeth on left valve. Umbo cavity not deep. Anterior adductor scar triangle.

Distribution and ecology: First record from Myanmar. The species is abundant in the lower reaches of the Kaladan and Lemro rivers. It inhabits a clay river bottom and was recorded from the boreholes of *Lignopholas fluminalis*, filled with clay. Local villagers actively harvest it for food and trade. In general, *Novaculina gangetica* seems to be an endemic species of the Ganges River basin in India and Bangladesh ranging from the delta to at least 1,500 km upstream[20,25,26], but molecular sequences of this lineage are not available. Our specimens from Myanmar may represent isolated populations of the latter taxon or a local endemic lineage, because the past connections between the Ganges and rivers of the western coast of Myanmar during the Pleistocene appear to be lacking[27]. The Kaladan River basin represents a separate evolutionary hotspot of fish diversity, with a number of endemic lineages[28], although the freshwater mussel fauna of this river system is poorly known[13,29].

Comments: We presented the first molecular data for a member of the genus. Our results indicate that *Novaculina* is most closely related to the *Sinonovacula* Prashad, 1924 (marine taxa) and *Pharella* Gray, 1854 (primarily marine and brackish taxa, with a single freshwater species[30]) (Fig. 5b).

Class Gastropoda Cuvier, 1795

Family Neritidae Rafinesque, 1815

Genus *Clithon* Montfort, 1810

Type species: *Nerita corona* Linnaeus, 1758 (by original designation)

Clithon cf. *reticularis* (Sowerby, 1838)

Material examined: Myanmar: Kaladan River, 21.0094° N, 92.9813° E, submerged siltstone rocks with boreholes, 01.v.2015, 30 specimens, Bolotov, Vikhrev, Aksenova, Konopleva, & local villagers leg.

Morphology: Shell finely striated, dark-yellow, with red and dark-red zigzag lines forming a marking pattern resembling fish scales. This pattern does not fit well with the typical *Clithon reticularis* having yellowish olive or green shell with oblique angulated red or black thin lines, often forming a coarse reticulation or network[22].

Distribution and ecology: This species is a common member of the rock-borer's community. The nominal taxon *Clithon reticularis* was described from Calcutta and it was also recorded from Parangipettai[22]. It is a brackish estuarine species, rarely extending into freshwater[22].

Comments: Kaladan's population seems to be a separate species-level lineage due to high morphological differences, but molecular sequences of *Clithon reticularis* from the type locality are not available.

Phylum Annelida Lamarck, 1809

Class Polychaeta Grube, 1850

Family Nereididae Blainville, 1818

Genus *Neanthes* Kinberg, 1865

Type species: *Neanthes vaalii* Kinberg, 1865 (by subsequent designation)

Neanthes meggitti (Monro, 1931)

Material examined: Myanmar: Kaladan River, 21.0094° N, 92.9813° E, submerged siltstone rocks with boreholes, 01.v.2015, 5 specimens, Bolotov, Vikhrev, Aksenova, Konopleva, & local villagers leg.

Morphology: The specimens comply with a recent re-description of *Neanthes meggitti* in most respects following important characteristics for delineation of nereidine species[31]. Paragnath numbers (Fig 4F, f2-f3) are within range of what was observed in type specimens, and parapodial characteristics confirm to the description of *N. meggitti*. A variation is observed in the collected specimens (Fig. 4F) compared to type material in the neuropodial postchaetal lobe which is longer and projecting beyond the acicular ligule compared to types which have a postchaetal lobe as long as acicular ligule[31].

Distribution and ecology: *Neanthes meggitti* was originally described from the Yangon River 64 km (40 miles) upstream from the city of Yangon[31]. Salinity conditions were not know for the sampling area in the Yangon River, but it was presumed have saltwater influence as the river was tidal at the sampling site[32]. It is also reported from the Hooghly estuary in west Bengal, on mud sediments among bricks and stones in the intertidal in the fresh water and low salinity zones[33].

Comments: The species was considered to be a freshwater species inhabiting rivers[34]. *N. meggitti* should also occur in lower tidal areas of rivers with some degree of salt water influence[32,33].

Genus *Namalycastis* Hartman, 1959

Type species: *Lycastis abiuma* Grube, 1872 (by original designation)

Namalycastis indica (Southern, 1921)

Material examined: Myanmar: Kaladan River, 21.0094° N, 92.9813° E, submerged siltstone rocks with boreholes, 01.v.2015, 6 specimens, Bolotov, Vikhrev, Aksenova, Konopleva, & local villagers leg.

Morphology: The specimens agree with both the original description of the species and the re-description of Glasby[35]. In particular, the slender, conical antennae and the long tentacular and anterior dorsal cirri set it apart from other *Namalycastis* species occurring in the region, such as *N. fauveli* Nageswara Rao, 1981 and *N. multiseta* Glasby, 1999. Further, the presence of a notochaetae in at least some parapodia and two pairs of equal-sized eyes sets the species apart from *N. glasbyi* Fernando & Rajasekaren, 2007 and *N. jaya* Magesh, Kvist & Glasby, 2012, both from southern India.

Distribution and ecology: The species occurs from India to Thailand in fresh to slightly brackish waters on muddy banks of rivers and backwater pools and lagoons[35]. This is the first record from a freshwater rock boring assemblage.

Phylum Porifera Grant, 1836

Class Demospongiae Sollas, 1885

Family Spongillidae Gray, 1867

Genus *Corvospongilla* Annandale, 1911

Type species: *Spongilla loricata* Weltner, 1895 (by subsequent designation)

Corvospongilla ultima (Annandale, 1910)

Material examined: Myanmar: Kaladan River, 21.0094° N, 92.9813° E, submerged siltstone rocks with boreholes, 01.v.2015, 2 specimens, Bolotov, Vikhrev, Aksenova, Konopleva, & local villagers leg.

Morphology: Sponge forming firm crusts on rocks (Fig. 3E); color in life deep green to brown. Skeleton alveolate, rather regular (Supplementary Figs. 3A & 3B). Megascleres (Supplementary Fig. 3C: c1) stout, feebly curved amphioxeas (minimum-<u>mean</u>-maximum values: 217-<u>242</u>-275 × 20-<u>28.5</u>-23.5 µm) which look smooth under LM, but very finely granulated under SEM (Supplementary Fig. 3C: c2). Microscleres (Supplementary Fig. 3C: c3) micropseudobirotules (minimum-<u>mean</u>-maximum values: 27.5-<u>37.5</u>-45 µm in length and to 2.5 µm in width) with smooth shaft and pseudorotules bearing 6-8 (rarely 4 or 5) large bent radial spines. Attached gemmules (Fig. 4E: e2) hemisperical, large (to 1 mm), adherent or sole, covered with a very dense cage made of microspined or smooth amphistrongyles (Supplementary Fig. 3C: c5) of variable size (130-35 µm) and megascleres. The cage forms a dark brown mat extending to several gemmules. Foramen single, lateral (Fig. 4E: e2), porus tube prominent (about 60 × 40 µm). Gemmuloscleres (Supplementary Fig. 3C: c4) spined amphistrongyles (minimum-<u>mean</u>-maximum values: 30.5-<u>43.5</u>-50 × 6-<u>7.3</u>-8.8 µm) tangentially arranged in the gemmular theca; spines of moderate size (to 1.3 µm), strait or slightly curved, distributed regularly or not. Pneumatic layer of fixed gemmules reduced. Free gemmules were not found in studied sponge fragments.

Distribution and ecology: First record for Myanmar. Previously known from freshwater bodies of India[36-45].

Comments: In the investigated sponge fragments only sessile gemmules occurred, which made impossible a study of pneumatic layer and the foramen structure of free gemmules. However, these traits contain no key diagnostic information at present[46]. Comparing to well-illustrated Indian specimens[37], specimens from the Kaladan River have some differences: more stout megascleres, longer microscleres, smaller gemmuloscleres' spines that are also often straight, and variable size of strongyles composing a gemmular cage. There is no crucial morphological distinction between the characters of investigated *C. ultima* and those of sponges studied by Jakhalekar and Ghate[37]. The original description[36], augmented by the revision of type material[47], also corresponds with the set of features found in *C. ultima* from Myanmar. Nevertheless, it is worth noting that as the knowledge on diversity and distribution of freshwater sponges in Southeast Asia is very poor[45], a real number of *Corvospongilla* species in this area, as well as true diagnostic significance of different characters, are far from being complete.

Supplementary References

1. Folmer, O., Black, M., Hoeh, W., Lutz, R. & Vrijenhoek, R. DNA primers for amplification of mitochondrial cytochrome c oxidase subunit I from diverse metazoan invertebrates. *Molecular Marine Biology and Biotechnology* **3**, 294–299 (1994).
2. Lobo, J. *et al.* Enhanced primers for amplification of DNA barcodes from a broad range of marine metazoans. *BMC Ecology* **13,** 34; DOI:10.1186/1472-6785-13-34 (2013).
3. Palumbi, S.R. Nucleic acids II: The polymerase chain reaction in *Molecular Systematics* (eds. Hillis D.M., Moritz C. & Mable B.K.) 205–247 (Sinauer Associates Inc., Sunderland, MA, 1996).
4. Lydeard, C., Mulvey, M. & Davis, G. M. Molecular systematics and evolution of reproductive traits of North American freshwater unionacean mussels (Mollusca: Bivalvia) as inferred from 16S *rRNA* gene sequences. *Philosophical Transactions of the Royal Society B: Biological Sciences* **351,** 1593–1603; DOI:10.1098/rstb.1996.0143 (1996).
5. Jovelin, R. & Justine, J. L. Phylogenetic relationships within the polyopisthocotylean monogeneans (Platyhelminthes) inferred from partial 28S rDNA sequences. *International Journal for Parasitology* **31,** 393–401; DOI:10.1016/S0020-7519(01)00114-X (2001).
6. Giribet, G., Carranza, S., Baguna, J., Riutort, M. & Ribera, C. First molecular evidence for the existence of a Tardigrada Arthropoda clade. *Molecular Biology and Evolution* **13**, 76–84 (1996).
7. Whiting, M.F., Carpenter, J.M., Wheeler, Q.D. & Wheeler, W.C. The Strepsiptera problem: phylogeny of the holometabolous insect orders inferred from 18S and 28S ribosomal DNA sequences and morphology. *Systematic Biology* **46**, 1–68; DOI:10.1093/sysbio/46.1.1 (1997).
8. WoRMS Editorial Board. *World Register of Marine Species* at http://www.marinespecies.org at VLIZ; DOI:10.14284/170 (2017).
9. Coan, E. V., Carlton, J. T. & Evenhuis, N. L. Case 3717 – Xylophagidae Purchon, 1941 (Mollusca: Bivalvia): proposed emendation of the spelling to Xylophagaidae to remove homonymy with Xylophagidae Fallén, 1810 (Insecta: Diptera). *The Bulletin of Zoological Nomenclature* **73**, 103–105; DOI:10.21805/bzn.v73i2.a4 (2017).
10. Combosch, D. J. & Giribet, G. Clarifying phylogenetic relationships and the evolutionary history of the bivalve order Arcida (Mollusca: Bivalvia: Pteriomorphia). *Molecular Phylogenetics and Evolution* **94,** 298–312; DOI:10.1016/j.ympev.2015.09.016 (2016).
11. Talavera, G. & Castresana, J. Improvement of phylogenies after removing divergent and ambiguously aligned blocks from protein sequence alignments. *Systematic Biology* **56,** 564–577; DOI:10.1080/10635150701472164 (2007).
12. Tamura, K., Stecher, G., Peterson, D., Filipski, A. & Kumar, S. MEGA6: Molecular Evolutionary Genetics Analysis version 6.0. *Molecular Biology and Evolution* **30,** 2725–2729; DOI:10.1093/molbev/mst197 (2013).
13. Bolotov, I.N. et al. Ancient river inference explains exceptional Oriental freshwater mussel radiations. *Scientific Reports* **7,** 2135; DOI:10.1038/s41598-017-02312-z (2017).
14. Blanford, W. T. Contributions of Indian Malacology, No. VIII. List of estuary shells collected in the delta of the Irawady, in Pegu, with descriptions of new species. *Journal of the Asiatic Society of Bengal* **36,** 51–72 (1867).

15. Turner, R. D. & Santhakumaran, L. N. The genera *Martesia* and *Lignopholas* in the Indo-Pacific (Mollusca: Bivalvia: Pholadidae). *Ophelia* **30,** 155–186; DOI:10.1080/00785326.1989.10430842 (1989).
16. Graf, D. L. Patterns of freshwater bivalve global diversity and the state of phylogenetic studies on the Unionoida, Sphaeriidae, and Cyrenidae. *American Malacological Bulletin* **31**, 135–153; DOI:10.4003/006.031.0106 (2013).
17. Chanley, P. E. Larval development of a boring clam, *Barnea truncata*. *Chesapeake Science* **6**, 162–166 (1965).
18. Boyle, P. J. & Turner, R. D. The larval development of the wood boring piddock *Martesia striata* (L.) (Mollusca: Bivalvia: Pholadidae). *Journal of Experimental Marine Biology and Ecology* **22**, 55–68; DOI:10.1016/0022-0981(76)90108-8 (1976).
19. Annandale, N. Bivalve molluscs injuring brickwork in Calcutta docks. *Journal of the Asiatic Society of Bengal* **18,** 555–557 (1922).
20. Nesemann, H., Sharma, G. & Sinha, R. Benthic macro-invertebrate fauna and "marine elements" sensu Annandale (1922) highlight the valuable dolphin habitat of river Ganga in Bihar-India. *Taprobanica* **3,** 18–30; DOI:10.4038/tapro.v3i1.3230 (2011).
21. Mienis H.K. & Rittner O. Brief review of the freshwater ark shells belonging to the genus *Scaphula* Benson, 1834 (Fam. Arcidae). *Triton* **34,** 16–20 (2016).
22. Subba Rao, N. V. *Handbook freshwater molluscs of India* (Zoological Survey of India, Calcutta, 1989).
23. Feng, Y., Li, Q. & Kong, L. Molecular phylogeny of Arcoidea with emphasis on Arcidae species (Bivalvia: Pteriomorphia) along the coast of China: Challenges to current classification of arcoids. *Molecular Phylogenetics and Evolution* **85**, 189–196; DOI:10.1016/j.ympev.2015.02.006 (2015).
24. Bogan, A.E. & Do, V.T. Two freshwater bivalve species new to the fauna of Vietnam (Mollusca: Bivalvia: Arcidae and Unionidae. *Tropical Natural History* **14,** 113–116 (2014).
25. Nesemann, H. A., Sharma, S. U., Sharma, G. O. & Sinha, R. K. Illustrated checklist of large freshwater bivalves of the Ganga river system (Mollusca: Bivalvia: Solecurtidae, Unionidae, Amblemidae). *Nachrichchtenblatt der Ersten Vorarlberger Malakologischen Gesellschaft* **13**, 1–51 (2005).
26. Baki, M. A., Hossain, M. M. & Bhouiyan, N. A. Checklist of freshwater mollusca (Gastropoda and Bivalvia) recorded from the Buriganga and Turag rivers, Dhaka, Bangladesh. *The Festivus* **48,** 221–228 (2016).
27. Voris, H. K. Maps of Pleistocene sea levels in Southeast Asia: shorelines, river systems and time durations. *Journal of Biogeography* **27,** 1153–1167; DOI:10.1046/j.1365-2699.2000.00489.x (2000).
28. Kottelat, M. The fishes of the inland waters of southeast Asia: a catalogue and core bibliography of the fishes known to occur in freshwaters, mangroves and estuaries. *Raffles Bulletin of Zoology, Supplement* **27,** 1–663 (2013).
29. Bolotov, I.N. et al. New taxa of freshwater mussels (Unionidae) from a species-rich but overlooked evolutionary hotspot in Southeast Asia. *Scientific Reports* **7,** 11573; DOI:10.1038/s41598-017-11957-9 (2017a).
30. Brandt, R. A. M. The non-marine aquatic Mollusca of Thailand. *Archiv fur Molluskenkunde* **105,** 1–423 (1974).
31. Bakken, T. Redescription of two species of *Neanthes* (Polychaeta: Nereididae) possessing a large notopodial prechaetal lobe. *Scientia Marina* **70S3**, 27–33; DOI:10.3989/scimar.2006.70s327 (2006).
32. Monro, C.C.A. A new brackish-water polychaete from Rangoon, *Nereis* (*Neanthes*) *megetti* sp. n. *Annals and Magazine of Natural History* **8**, 580–585 (1931).
33. Misra, A. Polychaete. Fauna of West Bengal in *State Fauna Series*, *Part 10* (ed. Gosh, A.K.) 125–225 (Zoological Survey of India, Calcutta, 1999).
34. Glasby, C.J., Timm, T., Muir, A.I. & Gil, J. Catalogue of non-marine Polychaeta (Annelida) of the World. *Zootaxa* **2070**, 1–52; DOI:10.5281/zenodo.187085 (2009).

35. Glasby, C.J. The Namanereidinae (Polychaeta: Nereididae). Part 1. Taxonomy and Phylogeny. *Records of the Australian Museum, Supplement* **25**: 1–129 (1999).
36. Annandale, N. Notes on Freshwater Sponges. XII. Description of a new species from Cape Comorin. *Records of the Indian Museum* **5**, 31 (1910).
37. Jakhalekar, S. S. & Ghate, H. V. Taxonomy of freshwater sponges of Maharashtra, India, with illustrated descriptions and notes on ecology and habitats (Porifera: Spongillida: Spongillidae).
38. Annandale, N. Freshwater sponges, hydroids and polyzoa. Porifera in *Fauna of British India, Including Ceylon and Burma* (ed. Shipley, A. E.) 241–245 (Taylor & Francis, London, 1911).
39. Annandale, N. The Freshwater Sponges of the Malabar zone. *Records of the Indian Museum* **7**, 383–397 (1912).
40. Soota, T. D. & Saxena, M. M. Sponge fauna of some waters of Rajasthan and its ecology. *Transactions of Indian Society of Desert Technology and University Centre of Desert Studies* **8**, 131–133 (1983).
41. Soota, T. D., Baskaran, S. & Saxena, M. M. Sponges of Lake Kailana, Jodhpur, Rajasthan and their ecology. *Geobios new Reports* **2**, 150–152 (1983).
42. Saxena, M. M. Freshwater sponges in the Thar Desert in *Faunal Diversity in the Thar Desert: Gaps in Research* (eds. Ghosh, A.K., Baqri, Q.H. & Prakash, I.) 37–41 (Scientific Publ., Jodhpur, 1996).
43. Manconi, R & Pronzato, R. Suborder Spongillina subord. nov.: Freshwater sponges in *Systema Porifera: A Guide to the Classification of Sponges, vol. 1* (eds. Hooper, J.N.A. & van Soest, R.W.M.) 921–1019 (Kluwer Academic/Plenum Publishers, New York, 2002).
44. Ruengsawang, N., Sangpradub, N., Hanjavanit, C. & Manconi, R. Biodiversity assessment of the Lower Mekong Basin: a new species of *Corvospongilla* (Porifera: Spongillina: Spongillidae) from Thailand. *Zootaxa* **3320**, 47–55 (2012).
45. Manconi, R. et al. Biodiversity in South East Asia: an overview of freshwater sponges (Porifera: Demospongiae: Spongillina). *Journal of Limnology* **72**, 313–326; DOI:10.4081/jlimnol.2013.s2.e15 (2013).
46. Manconi, R. & Pronzato, R. The genus *Corvospongilla* Annandale (Haplosclerida, Spongillina, Spongillidae) with description of a new species from eastern Mesopotamia, Iraq. *Archiv fur Hydrobiologie. Supplement Volumes, Monographic Studies*, **151**, 161–189 (2004).
47. Penney, J. T. & Racek, A. A. Comprehensive revision of a worldwide collection of freshwater sponges (Porifera: Spongillidae). *United State National Museum Bulletin* **272**, 1–184 (1986).
